# Enhancing privacy-preserving deployable large language models for perioperative complication detection: a targeted strategy with LoRA fine-tuning

**DOI:** 10.1038/s41746-025-02139-3

**Published:** 2025-12-13

**Authors:** Shaowei Gao, Xu Zhao, Lihui Chen, Junrong Yu, Shuning Tian, Huaqiang Zhou, Jingru Chen, Sizhe Long, Qiulan He, Xia Feng

**Affiliations:** 1 Department of Anesthesiology, First Affiliated Hospital of Sun Yat-sen University, Guangzhou, China; 2 Department of Anesthesiology, Jiangmen Central Hospital, Jiangmen, China; 3 Information and Data Center, First Affiliated Hospital of Sun Yat-sen University, Guangzhou, China; 4 Department of Medical Oncology, Sun Yat-sen University Cancer Center, Guangzhou, China

**Keywords:** Medical research, Health services

## Abstract

Perioperative complications are a major global concern, yet manual detection suffers from 27% under‑reporting and frequent misclassification. Clinical LLM deployment is constrained by data sovereignty, compute cost, and limited locally deployable model performance. We show targeted prompt engineering plus Low‑Rank Adaptation (LoRA) fine‑tuning converts smaller open‑source LLMs into expert‑level diagnostic tools. In dual‑center validation, we built a framework simultaneously identifying and grading 22 complication severities. State‑of‑the‑art models outperformed human experts; Chain‑of‑Thought prompting significantly improved general models (*p* < 0.001) while preserving reasoning models’ performance. Across documentation length quartiles, AI models maintained F1 > 0.64, whereas human performance declined from 0.73 to 0.45, demonstrating superior robustness to documentation complexity. Our targeted strategy—decomposing detection into focused single‑complication assessments—improved small models, with further gains from LoRA. On external validation (Center 2), the optimized 4B model’s micro‑F1 rose from 0.28 to 0.64, approaching human experts (F1 = 0.69), driven by the targeted strategy (ΔF1 = 0.256, 95% CI [0.181, 0.336]) and LoRA (ΔF1 = 0.103, 95% CI [0.023, 0.186]). Concurrently, the 8B model surpassed human experts (F1 > 0.70). Optimized small models enable expert‑level accuracy with local deployment and preserved data sovereignty, offering a practical path for resource‑limited healthcare.

## Introduction

Perioperative complications represent a major global health concern^1,2^, and have been recognized by the World Health Organization as a critical issue associated with patient suffering, reduced quality of life, and substantial healthcare costs^3^.

Timely and accurate identification of perioperative complications is vital for patient management, quality improvement, and surgical outcome assessment^4,5^. However, current clinical practice relies heavily on manual identification and documentation, methods fraught with significant drawbacks. Studies found 27% of complications, including serious events like occluded vascular grafts and bile leakage, missing from prospective registries with ~10% of reported events were misclassified^6^. Manual detection is error-prone, time-consuming, labor-intensive, and inconsistencies, comprising data reliability essential for quality improvement^5^.

Large Language Models (LLMs), built upon the transformer architecture^7^, have shown promising applications in areas such as clinical documentation assistance, medical question answering, and passing medical licensing examinations^8–11^. Furthermore, when deployed in perioperative care, their ability to provide automated screening and continuous (24/7) monitoring could transform perioperative quality management, especially during periods of reduced staffing when expert availability is limited.

However, application of commercial LLMs in healthcare faces critical barriers: (1) data sovereignty concerns under privacy regulations that restrict cloud-based inference^12,13^, (2) computational costs prohibiting many institutions from accessing large-parameter models^14^, and (3) infrastructure constraints requiring API calls that conflict with local data governance policies. These challenges necessitate smaller, locally-deployable models that maintain clinical accuracy.

We hypothesized that strategic task decomposition combined with parameter-efficient fine-tuning could enable smaller open-source models to achieve expert-level perioperative complication detection. We developed a “targeted strategy” structuring prompts around focused single-complication assessments, then applied Low-Rank Adaptation (LoRA)^15^ to fine-tune models (4B-32B parameters) using a dual-center dataset. This study demonstrates that optimized small models match or exceed human expert performance while maintaining local deployability, and provides open-source models, prompts, and evaluation framework to accelerate adoption in resource-constrained settings.

## Results

### Data and design

To evaluate LLM effectiveness in detecting postoperative complications from clinical records, this study utilized a dual-center design. Data were retrospectively collected from two independent medical centers: Center 1 (*n* = 146 cases) served as the primary dataset for model training and validation, while Center 2 (*n* = 102 cases) provided external validation.

The study cohort included patients undergoing diverse surgical procedures across multiple specialties (Table 1). Center 1 (*n* = 146) had a mean patient age of 57.7 ± 14.3 years and male predominance (65.8%), with surgical cases mainly from gastrointestinal (44.5%), hepatobiliary and pancreatic (32.9%), and urological (11.0%) surgery. Center 2 (*n* = 102) maintained similar demographic characteristics and diverse surgical specialties, featuring orthopedic (14.7%), gastrointestinal (20.6%), and urological (13.7%) surgery cases. The median length of hospital stay was 12 days (interquartile range (IQR): 9–15) for Center 1 and 10 days (IQR: 7–15) for Center 2. The most frequently observed complications were paralytic ileus (Center 1: 24.7%; Center 2: 19.6%), organ/space surgical site infections (14.4% vs. 15.7%), postoperative bleeding (6.8% vs. 14.7%), and acute kidney injury (4.8% vs. 11.8%). Notable variations in complication prevalence between centers reflected the diverse clinical contexts and patient populations inherent to multi-center healthcare settings.

**Table 1:** Patient Demographics and Clinical Characteristics by Center

Characteristic	Center 1	Center 2
Number of patients	146	102
Gender, *n* (%)		
Male	96 (65.8%)	62 (60.8%)
Female	50 (34.2%)	40 (39.2%)
Age, years (Mean ± SD)	57.7 ± 14.3	59 ± 13.7
Surgical specialty, n (%)		
Head and neck/other	4 (2.7%)	8 (7.8%)
Gynecological	5 (3.4%)	3 (2.9%)
Cardiovascular	5 (3.4%)	2 (2.0%)
Urological	16 (11.0%)	14 (13.70%)
Neurosurgery	3 (2.1%)	3 (2.9%)
Hepatobiliary and pancreatic	48 (32.9%)	17 (16.7%)
Gastrointestinal	65 (44.5%)	21 (20.6%)
Thoracic	0 (0.0%)	2 (2%)
Orthopedic	0 (0.0%)	15 (14.7%)
Median Length of hospital stay, days (IQR)	12 (9-15)	10 (7-15)
Complications, *n* (%)		
Paralytic ileus	36 (24.7%)	20 (19.6%)
Organ/space surgical site infection	21 (14.4%)	16 (15.7%)
Infection of unknown source	22 (15.1%)	3 (2.9%)
Myocardial injury after non-cardiac surgery	20 (13.7%)	7 (6.9%)
Anastomotic leakage	15 (10.3%)	10 (9.8%)
Pneumonia	10 (6.8%)	17 (6.7%)
Postoperative bleeding	10 (6.8%)	15 (14.7%)
Acute kidney injury	7 (4.8%)	12 (11.8%)
Superficial surgical site infection	4 (2.7%)	8 (7.8%)
Arrhythmia	7 (4.8%)	6 (5.9%)
Cardiogenic pulmonary edema	7 (4.8%)	1 (1.0%)
Deep vein thrombosis	5 (3.4%)	9 (8.8%)
Deep surgical site infection	3 (2.1%)	7 (6.9%)
Gastrointestinal bleeding	4 (2.7%)	6 (5.9%)
Delirium	5 (3.4%)	0 (0.0%)
Acute respiratory distress syndrome	4 (2.7%)	2 (2.0%)
Pulmonary embolism	3 (2.1%)	1 (1.0%)
Myocardial infarction	2 (1.4%)	1 (1.0%)
Bloodstream infection	1 (0.7%)	2 (2.0%)
Stroke	0 (0.0%)	1 (1.0%)

**Fig. 1:**
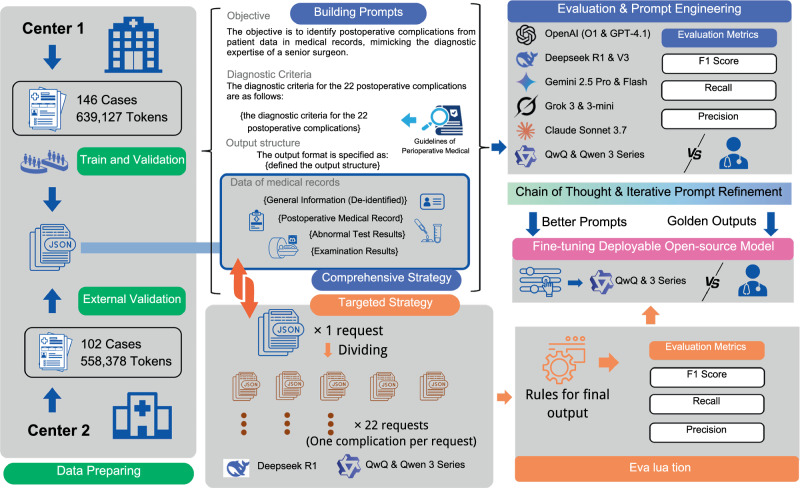
Methodological framework for AI-enhanced perioperative complication detection. The study utilized a dual-center design with data collection from Center 1 (*n* = 146 cases, 639,127 tokens) and Center 2 (*n* = 102 cases, 558,378 tokens). The workflow included: (1) prompt engineering using European Perioperative Clinical Outcome (EPCO) diagnostic criteria, (2) iterative evaluation and optimization across multiple AI models, and (3) comparison of comprehensive versus targeted strategies followed by LoRA fine-tuning of open-source models. Performance assessed using F1 score, recall, and precision metrics with comparative analysis between models and against human expert benchmarks. Figure created using Canva (www.canva.com).

For each case, we systematically extracted comprehensive clinical information including patient demographics, surgical procedure details, and postoperative findings—medical records, abnormal laboratory, and imaging results. Our methodological framework (Fig. 1) involved systematic prompt engineering and model selection, followed by iterative refinement driven by performance analysis, integrating both comprehensive and targeted strategies, culminating in LoRA fine-tuning of open-source models.

### Prompt development and initial evaluations

**Fig. 2:**
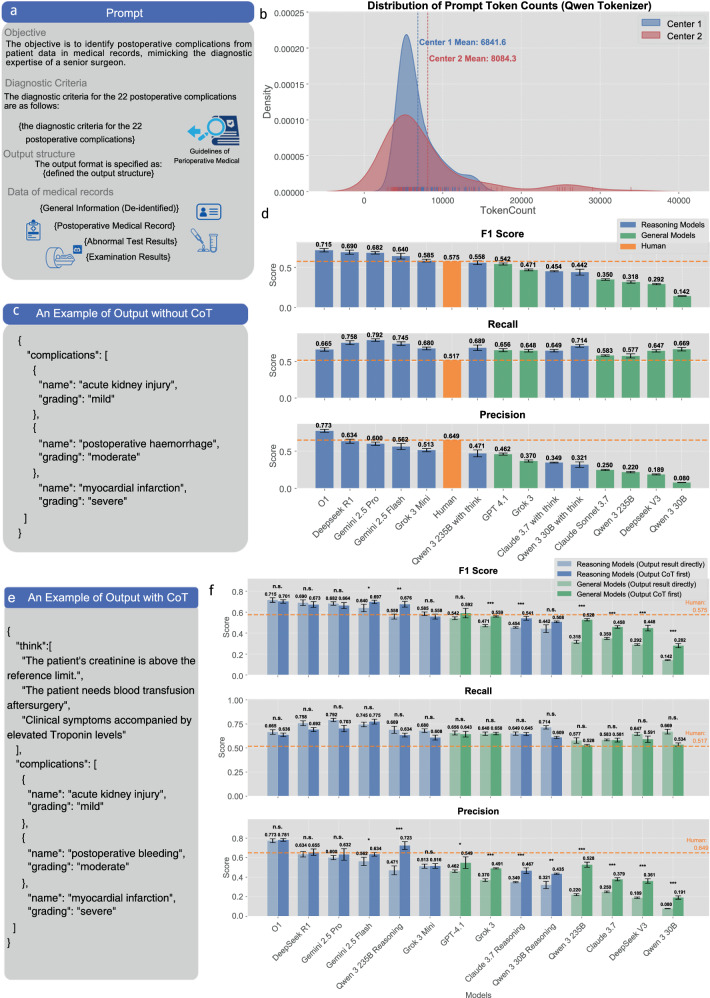
Prompt development and initial model evaluations. **a** Comprehensive prompt framework structure including objective definition, European Perioperative Clinical Outcome (EPCO) diagnostic criteria for 22 postoperative complications, structured JavaScript Object Notation (JSON) output format, and clinical data components (general information, postoperative medical records, abnormal test results, and examination results). **b** Token count distribution analysis revealing substantial heterogeneity between medical centers, with Center 2 exhibiting broader distribution patterns in clinical documentation length while Center 1 showed more concentrated distributions (Center 1 mean: 6841.6 tokens; Center 2 mean: 8084.3 tokens). **c** Example of basic structured JSON output format without Chain-of-Thought (CoT) prompting, showing direct complication identification and severity grading. **d** Initial performance evaluation across multiple state-of-the-art language models using micro-averaged metrics with confidence intervals from five repeated inferences (macro-averaged results in Supplementary Fig. 1), demonstrating superior performance of reasoning models over general models, with several AI models exceeding human expert benchmarks. **e** Example of CoT-enhanced JSON output incorporating diagnostic reasoning through the “think” field, enabling transparent clinical decision-making processes. **f** Performance comparison following CoT implementation using micro-averaged metrics with 95% confidence intervals from five repeated inferences and patient-level bootstrap paired testing (macro-averaged results in Supplementary Fig. 2; detailed statistics, effect sizes, and CIs in Supplementary Tables 1–2). Asterisks indicate statistical significance levels from bootstrap paired testing: * *p* < 0.05, ** *p* < 0.01, *** *p* < 0.001, showing significant improvements in general models while reasoning models maintained consistently high performance across F1 score, recall, and precision metrics. Figure created using Python matplotlib library; final composition assembled using Canva (www.canva.com).

Following our prompt construction methodology (see Methodology section; Figs. 1, 2a), we developed a comprehensive prompt framework enabling simultaneous identification and severity grading of 22 distinct perioperative complications—a notable improvement over traditional binary classification. Analysis of prompt token counts (Fig. 2b) revealed considerable heterogeneity between medical centers: Center 2 exhibited broader distribution patterns in clinical documentation length, while Center 1 showed more concentrated distributions (Center 1 mean: 6841.6 tokens; Center 2 mean: 8084.3 tokens). These markedly different distribution characteristics between centers created substantial heterogeneity, offering a rigorous test for model generalizability across diverse clinical documentation patterns.

Initial evaluations of multiple state-of-the-art LLMs using basic structured JavaScript Object Notation (JSON) output format (Fig. 2c) highlighted key performance trends (Fig. 2d, showing micro-averaged metrics with confidence intervals from five repeated inferences; macro-averaged results in Supplementary Fig. 1). Reasoning models consistently outperformed general models, with all models exceeding human performance belonging to the reasoning category. A notable difference in error patterns was observed: AI models generally demonstrated higher recall than precision (more sensitive but less specific), whereas human clinicians exhibited the converse (higher precision, lower recall). AI models were more comprehensive in detection, while human experts adopted more conservative diagnostic approaches.

### Primary prompt engineering

Based on initial evaluation results, we implemented Chain-of-Thought (CoT) prompting to improve diagnostic accuracy and model interpretability^16^. This evolved our structured JSON output from basic diagnostic formats (Fig. 2c) to CoT-enhanced responses (Fig. 2e), integrating reasoning transparency by adding a “think” field where models articulated diagnostic rationale before rendering final conclusions.

CoT implementation revealed distinct performance patterns across model architectures (Fig. 2f showing micro-averaged metrics with 95% confidence intervals from five repeated inferences and patient-level bootstrap paired testing; macro-averaged results in Supplementary Fig. 2; detailed statistics, effect sizes, and CIs in Supplementary Tables 1–2). General models demonstrated statistically significant performance enhancements with notable increases in F1 scores, precision, and recall (multiple models showing *p* < 0.001). Conversely, reasoning models showed only marginal and non-significant gains from explicit CoT prompting. This is consistent with their inherent design: reasoning models possess internal analytical processes, whereas general models derive substantial benefit from externally structured reasoning frameworks. Given statistically significant performance improvements in general models and maintained high performance in reasoning models, we adopted CoT prompting as the standard approach for all subsequent evaluations.

### Extended analysis and further prompt optimization

To deepen understanding of model behavior and further optimize performance, we undertook extended analysis encompassing stratified evaluation by document complexity and systematic identification of error patterns.

**Fig. 3:**
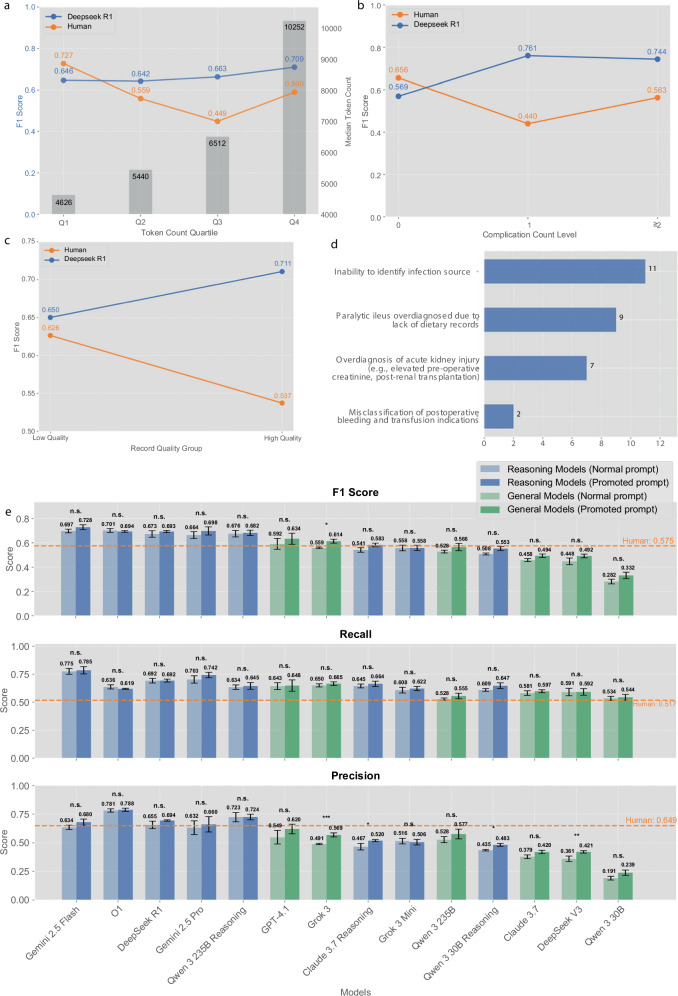
Extended analysis and prompt optimization results. **a** Stratified performance evaluation by document complexity, showing F1 scores across quartiles based on token counts (Q1: 4626 tokens, Q2: 5440 tokens, Q3: 6512 tokens, Q4: 10,252 tokens). DeepSeek R1 demonstrates consistent performance across document lengths, while human expert performance declines with increasing complexity. **b** Performance evaluation by case complexity based on perioperative complication count, showing F1 scores across three complexity levels (≤ 1 complication, 2 complications, and ≥3 complications). Human expert performance significantly declined as complication count increased, with F1 scores dropping from 0.656 to 0.440 to 0.563 in Center 1. AI models demonstrated remarkable consistency across all complexity levels, with DeepSeek R1 maintaining stable performance ranging from 0.569 to 0.744. **c** Performance evaluation by documentation quality using PDQI-9 scoring system, dividing cases into high-quality and low-quality groups. AI models demonstrated improved performance with higher-quality documentation (F1 = 0.711 vs. 0.650), while human experts showed inverse correlation (F1 = 0.537 vs. 0.626). **d** Error pattern analysis identifying primary categories of diagnostic failures, including inability to identify infection sources (11 cases), paralytic ileus overdiagnosis due to lack of dietary records (9 cases), overdiagnosis of acute kidney injury (7 cases), and misclassification of postoperative bleeding and transfusion indications (2 cases). **e** Performance comparison before and after prompt optimization across multiple models using micro-averaged metrics (macro-averaged results in Supplementary Fig. 3, detailed statistics, effect sizes, and CIs in Supplementary Tables 3–4), showing modest improvements in initially lower-performing models. Reasoning models (blue) and general models (green) are compared using normal prompts (lighter colors) versus optimized prompts (darker colors). Orange dashed lines indicate human expert benchmarks. Figure created using Python matplotlib library; final composition assembled using Canva (www.canva.com).

For stratified evaluation, cases were systematically divided into quartiles based on prompt token counts: Q1 (shortest documents, mean: 4626 tokens), Q2 (short-to-medium, mean: 5440 tokens), Q3 (medium-to-long, mean: 6512 tokens), and Q4 (longest and most complex documents, mean: 10,252 tokens). This analysis (Fig. 3a) revealed significant patterns in both human and AI performance. The top-performing model, DeepSeek R1, demonstrated remarkable consistency across document lengths, maintaining consistently high F1 scores irrespective of clinical documentation complexity. In contrast, human performance declined with increasing document length and complexity, suggesting that processing highly complex clinical records is challenging for human experts, potentially owing to cognitive load or fatigue^17,18^, whereas AI models exhibit superior scalability.

Complementing the document length analysis, we employed perioperative complication count as a direct metric of case complexity, stratifying cases into three levels: ≤1 complication, 2 complications, and ≥3 complications. Analysis revealed a critical differential pattern (Fig. 3b): human expert performance significantly declined as complication count increased, with F1 scores dropping from 0.656 (0 complications) to 0.440 (1 complication) and further to 0.563 (≥ 2 complications) in Center 1. In contrast, AI models demonstrated remarkable consistency across all complexity levels, with DeepSeek R1 maintaining stable performance ranging from 0.569 to 0.744 across the complexity spectrum. This pattern suggests AI systems are less susceptible to cognitive load associated with complex multi-complication cases.

Beyond case complexity, we systematically evaluated documentation quality using the PDQI-9 scoring system, dividing cases into high-quality and low-quality groups based on median scores. Remarkably, we observed contrasting sensitivity patterns between humans and AI (Fig. 3c): AI models demonstrated improved performance with higher-quality documentation (F1 = 0.711 for high-quality vs. 0.650 for low-quality), while human experts showed inverse correlation with documentation quality (F1 = 0.537 for high-quality vs. 0.626 for low-quality). This counterintuitive finding for human performance may reflect the positive correlation between documentation quality and text volume, suggesting human experts may be more sensitive to information overload in comprehensive documentation.

Systematic review of diagnostic errors identified primary categories of model failures (Fig. 3d), highlighting areas where clinical context interpretation needed refinement. We implemented prompt modifications (see Supplementary Information) incorporating four key enhancements:

1. *Enhanced Diagnostic Specificity*: We incorporated additional clinical exclusion criteria for several complications, such as specifying that acute kidney injury should not be diagnosed following kidney transplantation procedures, and providing explicit baseline creatinine calculation methods when preoperative values were unavailable.

2. *Documentation Clarification*: For paralytic ileus diagnoses, we added guidance regarding incomplete clinical documentation, stipulating that gastrointestinal symptoms (e.g., abdominal distension or vomiting) must be present when flatus or dietary records were incomplete.

3. *Differential Diagnostic Refinement*: We introduced important diagnostic distinctions for postoperative bleeding, specifically excluding cases where postoperative transfusions were administered despite minimal drainage output or pale drainage fluid, directing models to consider these as intraoperative blood loss or pre-existing anemia rather than true postoperative hemorrhage.

4. *Anatomical Precision Enhancement*: For anastomotic leakage, we expanded the definition to explicitly include external leakage, drainage site discharge, and imaging-identified extravasation, providing more comprehensive guidance for this critical complication.

These refinements yielded modest improvements across most model categories (Fig. 3e, showing micro-averaged metrics with 95% confidence intervals from five repeated inferences and patient-level bootstrap paired testing; macro-averaged results in Supplementary Fig. 3; detailed statistics, effect sizes, and CIs in Supplementary Tables 3–4). While most models demonstrated subtle improvement trends, the majority did not reach statistical significance for F1 and recall metrics (most comparisons n.s.). However, precision showed modest but statistically significant improvements in several models, including Qwen 3 30B Reasoning, Deepseek V3, and Grok 3 (*p* < 0.05 or *p* < 0.01). Notably, more pronounced improvements were consistently observed in models that initially exhibited lower baseline performance, suggesting that targeted prompt optimization can provide incremental benefits, particularly for underperforming models. These findings demonstrate that while prompt optimization offers measurable gains, the improvements are generally incremental rather than transformative, reflecting the inherent complexity of clinical diagnostic tasks and validating the appropriateness of using human expert performance as a realistic benchmark while highlighting the potential for AI assistance in complex clinical scenarios.

Although our analyses showed that leading commercial models could surpass human clinician performance, their practical implementation in healthcare settings requires addressing crucial concerns about data sovereignty, patient privacy, and computational cost-effectiveness. This necessity guided our investigation into the capabilities of open-source models for perioperative complication detection.

### Open-source AI performance

**Fig. 4:**
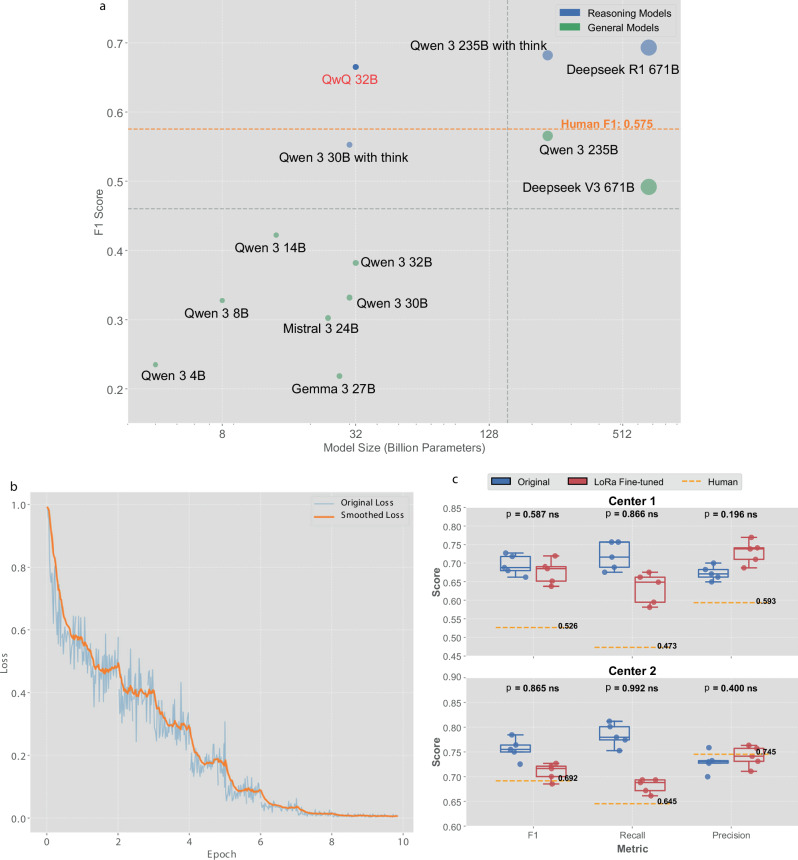
Open-source model performance evaluation and low-rank adaptation (LoRA) fine-tuning results. **a** Systematic evaluation of open-source models spanning 4B to 671B parameters using micro-averaged metrics (macro-averaged results in Supplementary Fig. 4). Point sizes are proportional to model parameter counts. The quadrant chart demonstrates correlation between model size and performance, with QwQ 32B achieving optimal balance between performance (F1 = 0.602) and efficiency while surpassing human expert benchmark (F1 = 0.575, orange dashed line). Reasoning models (blue) and general models (green) are distinguished by color coding. **b** LoRA fine-tuning convergence curves for QwQ 32B, showing original loss (blue) and smoothed loss (orange) trajectories across training epochs, demonstrating stable convergence within reasonable computational budgets. **c** Performance comparison before (blue) and after (red) LoRA fine-tuning across F1 score, recall, and precision metrics for both validation datasets using micro-averaged metrics and 95% confidence intervals from five repeated inferences with patient-level bootstrap paired testing (macro-averaged results in Supplementary Fig. 5; detailed statistics, effect sizes, and CIs in Supplementary Tables 5–6). Box plots show distribution of results with no statistically significant improvements observed (all *p* > 0.05). Orange dashed lines represent human expert benchmarks for each metric in respective centers. Figure created using Python matplotlib library; final composition assembled using Canva (www.canva.com).

We systematically evaluated open-source models from diverse manufacturers—including Qwen 3, DeepSeek, QwQ, Gemma, and Mistral—spanning parameter scales from 4B to 671B (Fig. 4a showing micro-averaged metrics from five repeated inferences; macro-averaged results in Supplementary Fig. 4). The quadrant chart reveals a clear correlation between model size and performance: larger parameter counts generally correspond to improved F1 scores. This relationship was not strictly linear, underscoring the significance of architectural design and training methodologies in addition to parameter scaling. Notably, QwQ 32B emerged as a top-performing open-source model, achieving an F1 score of 0.602 in the upper-left quadrant, demonstrating favorable balance between performance and efficiency while surpassing the human clinician benchmark (F1 = 0.575).

To further augment open-source model capabilities, we implemented LoRA fine-tuning on QwQ 32B, specifically targeting improved complication detection accuracy. The fine-tuning process (Fig. 4b) demonstrated convergence within reasonable computational budgets. However, performance improvements were modest across multiple evaluation metrics and on both validation data sources, yielding incremental gains rather than dramatic enhancements (Fig. 4c showing micro-averaged metrics and 95% confidence intervals from five repeated inferences with patient-level bootstrap paired testing; macro-averaged results in Supplementary Fig. 5; detailed statistics, effect sizes, and CIs in Supplementary Tables 5–6). This lack of improvement can be attributed to the model’s already high baseline performance, suggesting it had previously captured much of the clinical knowledge necessary for complication detection, leaving limited room for further enhancement through parameter-efficient fine-tuning. This motivated us to explore alternative approaches for performance enhancement.

Despite achieving near-human performance, practical deployment of 32B models exposed critical resource constraints. Clinical documents exceeding 15,000 tokens triggered out-of-memory errors even on high-end 80GB Graphics Processing Unit (GPU) systems, underscoring the urgent need for more efficient alternatives. Smaller models, conversely, grappled with the cognitive complexity of concurrently evaluating 22 distinct complications from extensive clinical narratives. These challenges motivated development of our targeted strategy: decomposing the comprehensive detection task into focused, single-complication assessments. Importantly, this strategic decomposition also functions as an innovative form of implicit data augmentation—transforming our cohort of 146 cases into approximately 3212 complication—specific examples (146 cases × 22 complications), thereby substantially enhancing the learning signal for our subsequent parameter-efficient fine-tuning methods, such as LoRA.

### Transition from comprehensive to targeted strategy

**Fig. 5:**
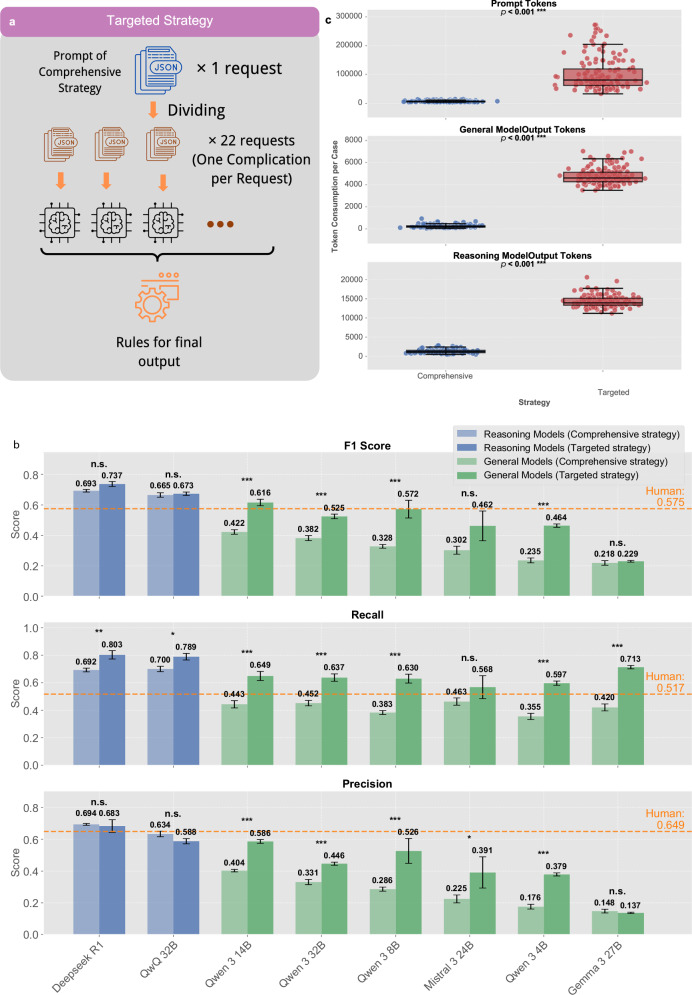
Transition from comprehensive to targeted strategy. **a** Schematic illustration of the targeted strategy approach, decomposing the comprehensive detection task (single request evaluating all 22 complications simultaneously) into 22 independent evaluation processes (one complication per request), followed by rules for generating final diagnostic output to ensure clinically coherent and mutually exclusive conclusions. **b** Performance comparison between comprehensive strategy (lighter colors) and targeted strategy (darker colors) across F1 score, recall, and precision metrics using micro-averaged results with patient-level bootstrap paired testing (macro-averaged results in Supplementary Fig. 6; detailed statistics, effect sizes, and CIs in Supplementary Tables 7-8). Reasoning models (light blue and blue) and general models (light green and green) demonstrate differential responses to strategy transition, with smaller models showing substantial and statistically significant improvements (Qwen3 4B-32B: all *p* < 0.001) while larger reasoning models maintain consistent performance. Orange dashed lines indicate human expert benchmarks for each metric. **c** Token consumption analysis comparing comprehensive versus targeted strategies across prompt tokens, general model output tokens, and reasoning model output tokens. Box plots show significant increases in computational requirements for the targeted approach (*p* < 0.001), representing the trade-off between enhanced accessibility for smaller models and increased computational volume. Figure created using Python matplotlib library; final composition assembled using Canva (www.canva.com).

Our targeted strategy (Fig. 5a) systematically divided the comprehensive detection task into 22 independent evaluation processes, requiring models to evaluate one specific complication per inference call rather than all 22 simultaneously. Each targeted prompt used identical clinical data inputs but was specifically designed to assess one complication type with focused diagnostic criteria and streamlined output requirements. Final diagnostic conclusions adhered to two key rules: (1) if all evaluations yielded negative results, the output was “no postoperative complications”; (2) identification of specific infectious complications automatically excluded a diagnosis of “infection of unknown source” to prevent redundancy. These rules (see “rule to get final output” in Fig. 5a) ensured clinically coherent and mutually exclusive diagnostic conclusions. This decomposition strategy maintained full diagnostic coverage while substantially reducing the cognitive load per inference call.

Comparing the two strategies revealed interesting performance patterns (Fig. 5b showing micro-averaged metrics with 95% confidence intervals from five repeated inferences and patient-level bootstrap paired testing; macro-averaged results in Supplementary Fig. 6; detailed statistics, effect sizes, and CIs in Supplementary Tables 7–8). Smaller general models demonstrated statistically significant improvements across all evaluation metrics with the targeted approach, with most models showing *p* < 0.001 for F1, recall, and precision. Notably, Qwen 3 4B, 8B, 14B, and 32B all achieved substantial performance gains, with F1 score improvements ranging from 0.19 to 0.24. In stark contrast, larger reasoning models exhibited minimal and non-significant performance changes when shifting strategies (both DeepSeek R1 and QwQ 32B showing n.s. across all metrics). Advanced reasoning models maintained nearly identical performance levels irrespective of strategic approach. This differential response suggests that larger, sophisticated models have sufficient computational capacity for effective comprehensive multi-complication analysis, whereas smaller models benefit considerably from cognitive load reduction inherent in targeted evaluation.

However, the targeted strategy substantially increases prompt token consumption per case, requiring multiple focused inferences for comprehensive diagnostic coverage (Fig. 5c). For commercial cloud-based models with per-token charges, this leads to significant cost implications. Conversely, this apparent computational drawback becomes a strategic advantage for local model deployment. In on-premises scenarios with fixed computational costs rather than usage-based charges, the targeted strategy allows smaller models to achieve performance levels previously attainable only by larger, more resource-intensive alternatives. This approach effectively trades increased computational volume for enhanced accessibility, enabling healthcare institutions to deploy smaller models on existing hardware while maintaining diagnostic accuracy.

### Fine tuning of small models

Building on the targeted strategy’s success in optimizing smaller models, we implemented LoRA fine-tuning to further enhance diagnostic capabilities of open-source models across various parameter scales. Our approach focused on four Qwen 3 models, ranging from 4B to 32B parameters, representing a spectrum suitable for diverse clinical environments.

**Fig. 6:**
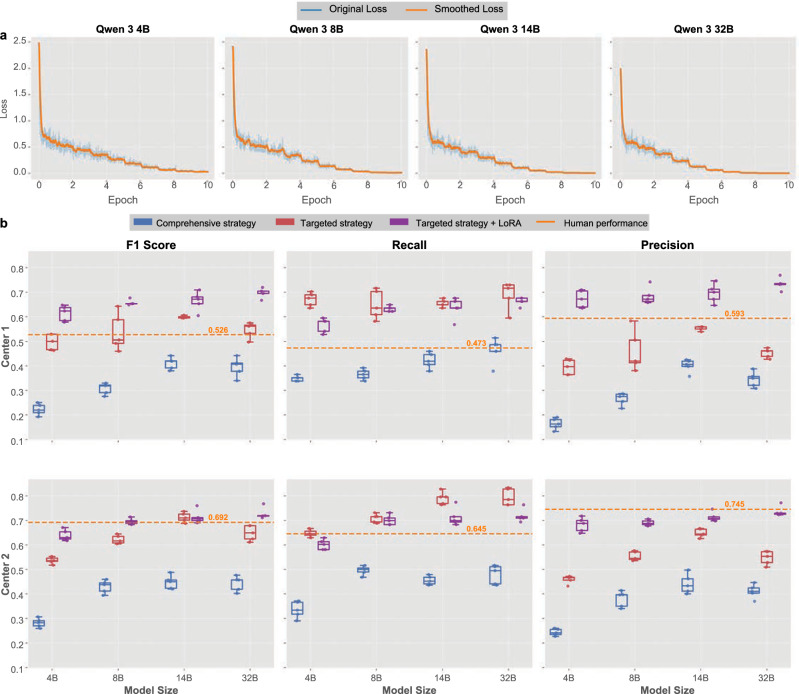
LoRA fine-tuning optimization results for small-scale open-source models. **a** Training convergence curves for Qwen 3 models across different parameter scales (4B, 8B, 14B, 32B), showing original loss (blue) and smoothed loss (orange) trajectories during LoRA fine-tuning process. All models demonstrate stable and consistent convergence patterns. **b** Comprehensive performance comparison across F1 score, recall, and precision metrics for both Center 1 and Center 2 validation datasets using micro-averaged metrics with 95% confidence intervals from five repeated inferences and patient-level bootstrap paired testing (macro-averaged results in Supplementary Fig. 7; detailed statistics, effect sizes, and CIs in Table 2 and Supplementary Table 9). Box plots illustrate performance distributions for comprehensive strategy (blue), targeted strategy (red), and targeted strategy with LoRA fine-tuning (purple), compared against human expert benchmarks (orange dashed lines). Results demonstrate that LoRA fine-tuning significantly enhances smaller models’ performance, with 4B and 8B parameter models showing substantial and statistically significant improvements. Figure created using Python matplotlib library; final composition assembled using Canva (www.canva.com).

The LoRA fine-tuning process (Fig. 6a) demonstrated consistent convergence patterns across all model sizes with smooth optimization trajectories. Fine-tuning results (Fig. 6b showing micro-averaged metrics with 95% confidence intervals from five repeated inferences; macro-averaged results in Supplementary Fig. 7) showed that LoRA adaptation substantially improved model performance across multiple evaluation metrics and on both validation data sources. In the Center 1 dataset, the 4B model showed dramatic enhancement: its F1 score increased from approximately 0.22 to 0.50 after targeted strategy implementation and further rose to 0.61 post-LoRA fine-tuning, passing the human performance benchmark of 0.526. The 8B model similarly demonstrated significant gains, achieving an F1 score above 0.66 after fine-tuning and surpassing human expert performance. Comparable trends were noted for model of other size and those in the Center 2 dataset.

To assess the statistical significance of these improvements, we employed patient-level bootstrap paired testing with 2000 resampling iterations and applied multiple comparison corrections (detailed statistical metrics with confidence intervals and effect sizes shown in Table 2 and Supplementary Table 9). For micro-averaged metrics, Center 1 validation showed strong statistical significance for the comprehensive vs targeted approach across all model sizes (*p* < 0.05 after Bonferroni correction). However, additional LoRA fine-tuning benefits were limited, with only the 32B model showing significant F1 improvement (ΔF1 = 0.149, 95% CI [0.021, 0.286], *p* = 0.021). For the expanded Center 2 external validation cohort (*n* = 102), all models demonstrated highly significant improvements with the comprehensive vs targeted approach (*p* < 0.001). The 4B and 8B models showed additional statistically significant improvements with Targeted+LoRA: ΔF1 = 0.103 (95% CI [0.023, 0.186], *p* = 0.011) for 4B and ΔF1 = 0.073 (95% CI [0.002, 0.144], *p* = 0.046) for 8B after Bonferroni correction. Macro-averaged results demonstrated similar patterns with subtle variations in effect magnitudes (Supplementary Table 9).

**Table 2:** Statistical Analysis of Model Performance Improvements with Bootstrap Paired Testing

Center	Model	Comparison	Metric	Mean Diff	95% CI	*p*-value	*p*-corrected
Center1 (*n* = 46)	4B	Comprehensive vs Targeted	F1	0.273	[0.151, 0.385]	**<0.001**	**0.001**
			Precision	0.229	[0.119, 0.330]	**<0.001**	**0.001**
			Recall	0.321	[0.147, 0.480]	**<0.001**	**0.001**
		Targeted vs Targeted + SFT	F1	0.116	[−0.022, 0.255]	0.0535	0.107
			Precision	0.277	[0.137, 0.414]	**<0.001**	**0.001**
			Recall	−0.104	[−0.263, 0.060]	0.9055	1.000
	8B	Comprehensive vs Targeted	F1	0.229	[0.072, 0.420]	**0.0015**	**0.003**
			Precision	0.194	[0.040, 0.395]	**0.002**	**0.004**
			Recall	0.283	[0.103, 0.468]	**0.0015**	**0.003**
		Targeted vs Targeted + SFT	F1	0.117	[−0.066, 0.278]	0.110	0.220
			Precision	0.221	[0.022, 0.398]	**0.0155**	**0.031**
			Recall	−0.017	[−0.186, 0.150]	0.602	1.000
	14B	Comprehensive vs Targeted	F1	0.188	[0.054, 0.321]	**0.0025**	**0.005**
			Precision	0.150	[0.006, 0.294]	**0.0215**	**0.043**
			Recall	0.231	[0.077, 0.386]	**0.0015**	**0.003**
		Targeted vs Targeted + SFT	F1	0.066	[−0.073, 0.197]	0.1685	0.337
			Precision	0.145	[0.007, 0.276]	**0.0225**	**0.045**
			Recall	−0.017	[−0.174, 0.139]	0.618	1.000
	32B	Comprehensive vs Targeted	F1	0.153	[0.017, 0.293]	**0.0165**	**0.033**
			Precision	0.110	[−0.011, 0.235]	**0.043**	0.086
			Recall	0.228	[0.027, 0.410]	**0.0185**	**0.037**
		Targeted vs Targeted + SFT	F1	0.149	[0.021, 0.286]	**0.0105**	**0.021**
			Precision	0.280	[0.156, 0.410]	**<0.001**	**0.001**
			Recall	−0.027	[−0.179, 0.153]	0.6765	1.000
Center 2 (*n* = 102)	4B	Comprehensive vs Targeted	F1	0.256	[0.181, 0.336]	**<0.001**	**0.001**
			Precision	0.216	[0.142, 0.290]	**<0.001**	**0.001**
			Recall	0.311	[0.201, 0.422]	**<0.001**	**0.001**
		Targeted vs Targeted + SFT	F1	0.103	[0.023, 0.186]	**0.0055**	**0.011**
			Precision	0.224	[0.133, 0.313]	**<0.001**	**0.001**
			Recall	−0.046	[−0.142, 0.054]	0.8375	1.000
	8B	Comprehensive vs Targeted	F1	0.192	[0.094, 0.296]	**<0.001**	**0.001**
			Precision	0.173	[0.053, 0.292]	**0.001**	**0.002**
			Recall	0.212	[0.114, 0.315]	**<0.001**	**0.001**
		Targeted vs Targeted + SFT	F1	0.073	[0.002, 0.144]	**0.023**	**0.046**
			Precision	0.136	[0.061, 0.210]	**<0.001**	**0.001**
			Recall	-0.008	[-−0.097, 0.085]	0.605	1.000
	14B	Comprehensive vs Targeted	F1	0.267	[0.163, 0.366]	**<0.001**	**0.001**
			Precision	0.208	[0.088, 0.317]	**<0.001**	**0.001**
			Recall	0.338	[0.229, 0.451]	**<0.001**	**0.001**
		Targeted vs Targeted + SFT	F1	0.001	[−0.071, 0.086]	0.5155	1.000
			Precision	0.067	[−0.008, 0.145]	**0.040**	0.080
			Recall	-0.078	[−0.169, 0.033]	0.920	1.000
	32B	Comprehensive vs Targeted	F1	0.207	[0.107, 0.313]	**<0.001**	**0.001**
			Precision	0.137	[0.032, 0.242]	**0.002**	**0.004**
			Recall	0.316	[0.195, 0.439]	**<0.001**	**0.001**
		Targeted vs Targeted + SFT	F1	0.079	[−0.007, 0.172]	**0.042**	0.084
			Precision	0.188	[0.104, 0.277]	**<0.001**	**0.001**
			Recall	-0.076	[−0.176, 0.037]	0.914	1.000

Statistical Methods: Patient-level bootstrap paired testing with 2000 iterations. All tests are single-sided (greater than) with Bonferroni correction for multiple comparisons.

Note: Positive values indicate improvement in the second strategy compared to the first.

*SFT* Supervised Fine-Tuning (LoRA), *CI* Confidence Interval, *Mean Diff* Mean Difference.

Bold values indicate statistical significance at *p*<0.05 after Bonferroni correction.

To foster research transparency and stimulate further advancements, our fine-tuned models have been made publicly available on the Hugging Face platform with the suffix “-PeriComp” appended to their original names (https://huggingface.co/collections/gscfwid/pericomp). Open-sourcing these specialized models aims to empower other researchers and drive further breakthroughs in clinical AI.

### Strict performance

To further validate clinical utility of our optimized models and rigorously compare our fine-tuned models with leading commercial/cloud counterparts, we implemented a more stringent evaluation protocol. This strict assessment mandated complete diagnostic accuracy: correct identification of both the specific complication type and its severity grade (mild, moderate, or severe) was required for a diagnosis to be deemed valid.

**Fig. 7:**
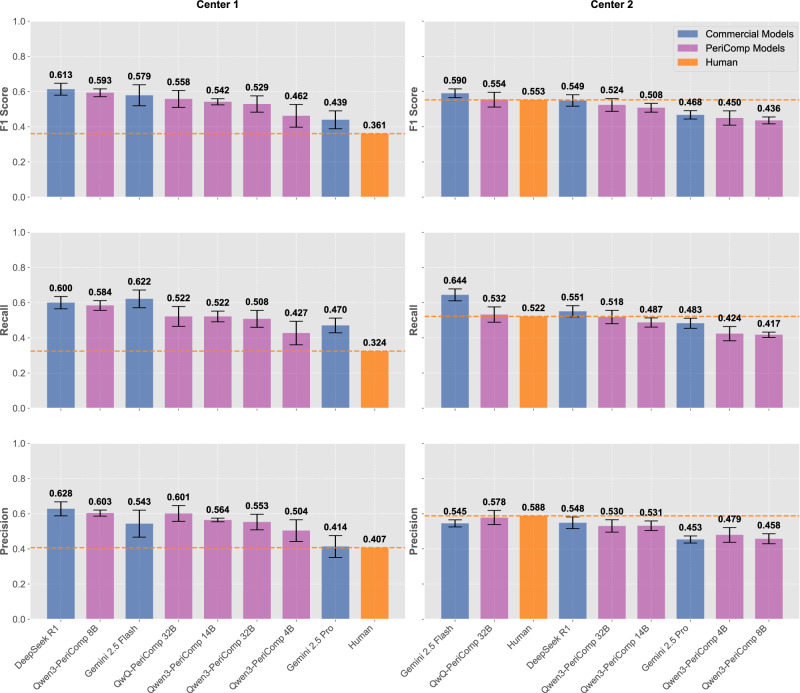
Strict performance evaluation with complete diagnostic accuracy requirements. Comprehensive performance comparison across F1 score, recall, and precision metrics for both Center 1 and Center 2 validation datasets using micro-averaged metrics with 95% confidence intervals from five repeated inferences (macro-averaged results in Supplementary Fig. 8). Evaluation under strict criteria requires correct identification of both specific complication type and severity grade (mild, moderate, or severe) for a diagnosis to be considered valid. Commercial models (blue) including Gemini 2.5 Flash, DeepSeek R1, and Gemini 2.5 Pro are compared against fine-tuned PeriComp models (purple) including QwQ-PeriComp-4B, QwQ-PeriComp-8B, QwQ-PeriComp-14B, and QwQ-PeriComp-32B, with human expert performance (orange) serving as clinical benchmarks. Orange dashed lines indicate human expert performance thresholds for each metric in respective centers. Figure created using Python matplotlib library.

The strict performance evaluation (Fig. 7 showing micro-averaged metrics with 95% confidence intervals from five repeated inferences; macro-averaged results in Supplementary Fig. 8) revealed key insights into model capabilities and methodological approach efficacy. Although absolute scores were lower across all models compared to non-strict evaluations, the performance of our fine-tuned models substantially approached or exceeded that of leading commercial/cloud models and human experts across both validation datasets.

## Discussion

This study advances perioperative complication detection through several key innovations. We introduced a novel prompt framework enabling simultaneous identification and severity grading via structured output, demonstrating AI’s capacity to match or surpass human expert performance. Comprehensive evaluations identified QwQ 32B as achieving optimal balance between performance and deployment efficiency. To improve accessibility and efficacy of smaller models, we first implemented a task decomposition strategy (targeted strategy), which markedly elevated their capabilities to expert level even before fine-tuning. Subsequent LoRA fine-tuning of compact models (4B–32B parameters), leveraging this decomposed framework, further consolidated and augmented this expert-level performance, ensuring they could rival or exceed human expertise while maintaining practical deployability. Our optimized models and code have been open-sourced to foster advancements in perioperative AI.

Our task decomposition strategy aligns with established principles in machine learning, notably in modular learning and prompt engineering. Recent progress in decomposed prompting has shown substantial improvements in complex reasoning tasks by segmenting problems into manageable, sequentially solvable subtasks^19^. This modular methodology has been validated in neuro-symbolic learning frameworks, where systematic decomposition benefits complex reasoning tasks more than end-to-end learning^20^. Our clinical application of this principle reconfigures cognitive load distribution from concurrent multi-task processing to sequential single-task optimization. This shift enables sophisticated diagnostic capabilities in resource-limited models, maintaining performance comparable to that of larger, more computationally intensive alternatives.

Previous research laid important foundations for AI applications in perioperative medicine. Traditional machine learning approaches demonstrated effectiveness across specific complications or narrow procedural contexts, typically yielding binary prediction outputs^21–24^. Recent LLM studies showed promise: Chung et al. (2024) examined GPT-4 Turbo’s capabilities in perioperative risk prediction using real-world electronic health records^8^, while Hsueh et al. (2024) investigated GPT-4’s capacity to detect postoperative complications from renal surgery discharge summaries^4^. However, both studies relied exclusively on commercial cloud-based models and did not explore systematic performance enhancement strategies, posing significant barriers to clinical deployment arising from data sovereignty, patient privacy, and computational cost concerns. Established perioperative risk assessment tools such as POSSUM and P-POSSUM scoring systems and the ACS-NSQIP database require manual data entry and typically focus on limited outcome categories^25^.

In contrast, our study presents a comprehensive framework addressing these limitations via iterative model enhancement. Through targeted task decomposition and LoRA fine-tuning, we systematically optimized model performance in processing unstructured clinical narratives directly. Our approach facilitates simultaneous detection and severity grading across 22 distinct perioperative complication types based on European Perioperative Clinical Outcome (EPCO) definitions—achieving complexity and coverage that surpasses prior work. Compared to existing approaches, our system offers automated extraction from unstructured clinical text rather than manual data entry, comprehensive screening with severity grading, and real-time postoperative monitoring capability that can run on single GPU servers within hospital networks, maintaining data sovereignty. More critically, our targeted strategy significantly boosts performance of smaller, resource-efficient models, enabling them to match or exceed both human expert capabilities and commercial model benchmarks while remaining deployable within existing healthcare infrastructure.

The observed performance disparities among open-source models (Fig. 4a) reflect the interplay between language-specific training data and architectural constraints. Our exclusive use of Chinese medical records naturally favored models with substantial Chinese language representation in their pre-training corpora. For instance, Qwen models, designed with significant Chinese content (approximately 22.7% Chinese text data per Bai et al., 2023)^26^, outperformed Gemma and Mistral models, which were predominantly trained on English-centric datasets with minimal Chinese representation. This language alignment effect is well-documented in multilingual model research, where performance correlates strongly with the quantity and quality of language-specific training data^27^ and aligns with cross-lingual medical NLP findings where teams achieved optimal performance by matching pre-trained models to their target language—Japanese medical tasks benefited from UTH-BERT and Japanese medical models, while English tasks performed best with BioBERT, ClinicalBERT, and PubMedBERT^28^. Concurrently, architectural constraints—particularly context length limitations—are equally important: many of our medical documents exceed 10,000 tokens, and models with insufficient context windows face fundamental processing constraints regardless of language considerations. For cross-linguistic deployment, our targeted strategy’s task decomposition approach is fundamentally language-agnostic, reducing cognitive load regardless of underlying language. Implementation would likely require: (1) fine-tuning with target-language perioperative data, (2) selection of models with substantial target-language pre-training representation and adequate context length capabilities, and (3) localization of prompt frameworks incorporating culture-specific medical terminologies and diagnostic criteria. We hypothesize that similar performance gains would be achievable with English-primary models (e.g., Llama, Mistral) fine-tuned on English perioperative records, though future replication studies with diverse language datasets are necessary confirm methodology transferability across linguistic contexts.

Our sensitivity analysis (Fig. 3) revealed fundamental differences in how humans and AI models respond to clinical complexity challenges, providing crucial insights for understanding the marked heterogeneity in human expert performance observed in our strict evaluation (Fig. 7). Systematic evaluation across multiple complexity dimensions—document length, complication count, and documentation quality—demonstrated divergent performance patterns: AI models maintained consistent diagnostic accuracy regardless of these variables, while human experts exhibited systematic performance degradation with increasing document complexity and complication burden, paradoxically showing inverse correlation with documentation quality. These contrasting patterns likely reflect cognitive overload affecting human experts when processing comprehensive clinical narratives, whereas AI models showed stable or improved performance across all complexity levels, with enhanced accuracy in high-quality documentation scenarios^17,18^.

These differential sensitivity patterns directly explain observed inter-center disparities and inform clinical implementation strategies. In the strict evaluation, experts attained F1 scores below 0.4 at Center 1, in stark contrast to the significantly higher scores observed at Center 2. This disparity reflects systematic differences in case complexity: Center 1 demonstrated higher complexity with fewer zero-complication cases (34.25% vs. 42.16%) and proportionally more multi-complication cases. The complementary sensitivity patterns between humans and AI suggest several implementation considerations: tertiary referral centers handling complex cases may derive greater benefit from AI assistance where human performance typically degrades; documentation quality sensitivity patterns support adaptive AI systems that adjust confidence thresholds based on completeness; and hybrid human-AI diagnostic workflows could optimize resource allocation by prioritizing AI assistance for complex multi-complication cases while leveraging the complementary strengths of both approaches.

Several limitations warrant acknowledgment. First, while our study exclusively utilized Chinese-language clinical records, this limitation affects direct model deployment rather than the underlying methodological framework. The targeted strategy and LoRA fine-tuning methodology demonstrate broad applicability, though cross-linguistic implementation would require language-specific fine-tuning with target-language datasets. Additionally, our study did not include explicit demographic bias analysis, which represents an important limitation for clinical deployment. Potential algorithmic biases could manifest through underrepresentation of specific demographic groups in training data, cultural nuances in clinical documentation patterns, or differential complication presentation across patient subpopulations. Future implementations must incorporate systematic demographic stratification during validation and establish bias monitoring protocols to ensure equitable performance across diverse patient populations.

Second, our approach faces fundamental constraints related to clinical documentation quality that extend beyond technical model limitations. While our sensitivity analysis using PDQI-9 scoring demonstrated that AI performance improves with higher-quality documentation, we must acknowledge an inherent ceiling on diagnostic accuracy: when critical clinical information is omitted at the source—such as when physicians fail to document patient symptoms, examination findings, or laboratory abnormalities—even expert human clinicians reviewing the same documentation would be unable to reconstruct the patient’s actual clinical state. This limitation affects both human and AI-based approaches when working from incomplete clinical records, representing a fundamental bottleneck that no diagnostic system can overcome without addressing documentation quality at its source.

Third, our training cohort of 100 cases, while substantial compared to the sample sizes proven effective for LoRA-based medical AI applications (literature reports successful LoRA fine-tuning with as few as 2-32 samples per class^29^ and 16 samples per class for medical vision-language models^30^, represents a relatively modest dataset by traditional deep learning standards. This limitation is partially mitigated by our targeted strategy’s data augmentation effect, which transforms each case into 22 complication-specific examples, and by LoRA’s theoretical advantages for smaller datasets. However, our training dataset encompasses 19 out of 22 EPCO-defined complications, potentially limiting model performance on the three rare complications not represented in our cohort. As an exploratory study, future implementations across different institutions may require larger, more comprehensive datasets to ensure robust performance across the full spectrum of perioperative complications.

Fourth, our training cohort, despite clinical diversity, did not encompass the complete spectrum of surgical procedures and complication patterns in contemporary perioperative practice. Future research incorporating more comprehensive and varied datasets—spanning additional surgical specialties, rare complications, and diverse institutional practices—could potentially yield further performance enhancements and bolster the robustness of our diagnostic framework.

Building upon our findings, we propose key directions for clinical implementation and validation. Institutions implementing similar systems should consider minimum training cohorts of 150-200 cases, leveraging federated learning for multi-institutional collaboration that achieves optimal dataset sizes while preserving data sovereignty. Our LoRA fine-tuning approach enables local deployment on institutional infrastructure, maintaining computational efficiency and patient privacy without cloud-based inference requirements. Technical implementation requires electronic health record system integration with adaptive quality assurance frameworks incorporating automated PDQI-9 scoring and alert management strategies, while our structured output format with Chain-of-Thought reasoning enables clinicians to review diagnostic conclusions and underlying rationale, positioning AI predictions as supplementary assessments rather than definitive diagnoses.

Prospective validation represents the essential next step toward clinical deployment, requiring systematic evaluation in real-world environments through scheduled assessment protocols monitoring performance without initially influencing clinical decisions. We propose phased implementation: retrospective validation, followed by silent monitoring, then graduated deployment with progressive alert introduction based on reasoning quality assessment and user feedback. This implementation framework must incorporate systematic bias assessment through demographic stratification protocols and continuous monitoring across patient subgroups to ensure equitable performance. Continuous improvement could be achieved through systematic collection of clinical outcomes and expert feedback, with our open-source release facilitating collaborative enhancement across institutions. Beyond technical considerations, successful deployment must address organizational readiness, clinician trust, and liability frameworks for AI-assisted decisions. Long-term, AI-assisted documentation systems could improve documentation quality at its source, creating positive feedback loops where enhanced documentation enables more accurate AI diagnosis.

In conclusion, this study signifies notable advancement in perioperative complication detection by establishing a comprehensive framework that marries cutting-edge AI capabilities with practical clinical deployment constraints. Our innovative amalgamation of targeted strategy and LoRA fine-tuning democratizes expert-level diagnostic performance, enabling resource-limited healthcare institutions to deploy sophisticated AI systems using modest computational infrastructure. Through open-sourcing our fine-tuned models and comprehensive prompt templates, supported by rigorous dual-center validation, we offer a foundational platform for future research and clinical implementation, providing a blueprint for developing specialized medical AI systems that balance performance, accessibility, and deployment considerations.

## Methods

### Study design and data collection

This study utilized a dual-center design to assess LLM performance in detecting postoperative complications from clinical narratives. Conducted in adherence to ethical principles, the research received approval from institutional review boards of both participating medical centers (First Affiliated Hospital of Sun Yat-sen University Ethics Committee Approval: No. [2025] 019; Jiangmen Central Hospital Ethics Committee Approval: No. [2025] 180 A). Given the retrospective nature of data collection, informed consent was waived by the ethics committees. The dual-center approach was chosen to evaluate model generalizability across varied healthcare environments and patient populations with distinct complexity profiles.

Center 1, a regional tertiary medical center, is characterized by complex cases, extensive clinical documentation, and a broad spectrum of postoperative complications. It serves as a referral hub for challenging surgical cases, which typically involve longer clinical narratives and more diverse complication patterns. In contrast, Center 2 is a secondary-level hospital exhibiting characteristics typical of community healthcare settings, such as relatively straightforward cases and concise documentation.

Inclusion criteria were formulated to capture a representative sample of complex surgical cases with adequate postoperative observation periods. Specifically, eligible patients were aged >14 years; for Center 1, an additional criterion mandated postoperative hospital stays exceeding 7 days to ensure appropriate case complexity. No restrictions were imposed on surgical specialty or procedure type.

Following the European Perioperative Clinical Outcome (EPCO) framework^31^, we use “perioperative complications” to describe the 22 postoperative adverse events evaluated in this study (occurring from end of surgery through discharge or postoperative day 30).

Electronic health records from the two independent medical centers, covering January 2023 to February 2025, were systematically extracted. For every selected case, four core data components were systematically extracted: (1) demographic and procedural information, (2) postoperative medical records, including daily clinical notes and nursing documentation, (3) abnormal postoperative laboratory results, and (4) postoperative imaging and examination reports in narrative text format.

Following systematic extraction and exclusion criteria application, the final cohort consisted of 146 cases from Center 1 and 102 cases from Center 2, totaling 248 cases for subsequent model evaluation and enhancement.

A portion of Center 1 data was strategically utilized for model fine-tuning, with the remainder of Center 1 cases and all Center 2 cases reserved for validation. Specifically, the 146 cases from Center 1 were randomly divided into a training cohort (*n* = 100) for fine-tuning and a validation cohort (*n* = 46) for performance assessment. All 102 cases from Center 2 were allocated for external validation to evaluate model generalizability. Given the heterogeneous characteristics between the two centers, we used only Center 1 data for comprehensive model comparisons and fine-tuning processes, while Center 2 data was reserved exclusively for external validation to ensure robust assessment of model generalizability.

### Data preprocessing and anonymization

All clinical narratives were standardized into a consistent markdown format to ensure uniform prompt integration. This structured methodology organized information hierarchically, encompassing general patient information, chronological postoperative medical records, abnormal laboratory findings, and examination reports.

Comprehensive data anonymization was performed to safeguard patient privacy. All direct identifiers were systematically removed. For temporal data, a sophisticated time-shifting methodology was implemented. We applied a unique, randomly generated temporal offset (in days) to each patient’s time-related data. This offset, inspired by established practices in medical databases like MIMIC^32^, was uniformly applied to all fields, effectively preserving the relative temporal relationships and intervals between clinical events.

### Prompt engineering framework

The prompt architecture designated the LLM as an experienced surgeon with specialized expertise in perioperative medicine, with objective explicitly defined as systematic identification and severity grading of postoperative complications based on clinical documentation and standardized diagnostic criteria.

The EPCO definitions framework was selected over alternative systems, such as the Clavien-Dindo classification^31,33^. This framework delineates 22 distinct perioperative complications—most stratified into mild, moderate, and severe categories—providing comprehensive coverage with specific diagnostic criteria for each complication type.

A rigorous JSON schema-based output format, consistent with current OpenAI Software Development Kit standards, was implemented (as exemplified in Fig. 2c, e). Each complication was structured as an object with two primary attributes: complication type and severity grade. All identified complications were organized into a standardized list, with “no postoperative complications” serving as the single-element response if no complications were detected.

As a subsequent optimization strategy, we evaluated CoT prompting incorporation to enhance diagnostic transparency and reasoning validation. When implemented, CoT-enhanced prompts included a “think” object containing structured reasoning elements.

A complete example of the structured prompt template is available in Supplementary Information for reference and reproducibility.

### Model selection and deployment architecture

The evaluation encompassed state-of-the-art commercial models including general and reasoning models from OpenAI, Anthropic, Google, Grok, and DeepSeek^34,35^. Open-source models evaluated included DeepSeek R1 & V3, QwQ 32B, the Qwen 3 series (4B, 8B, 14B, 32B), Gemma 3 (27B), and Mistral 3 Small 24B^36,37^. Detailed version specifications for all models are provided in Supplementary Table 10.

Commercial models and large open-source models were accessed via established Application Programming Interface (API) services. Smaller open-source models were deployed locally on computational nodes at the National Supercomputer Center in Guangzhou, using high-performance A800 (80GB) GPU clusters with 1–4 GPUs employed in parallel configuration.

We employed MS-Swift as our fine-tuning framework for LoRA implementation^38^. Key LoRA parameters were optimized: lora_rank=16, lora_alpha=32, and learning_rate=1e-4. Local model deployment and inference utilized vLLM with integrated Outlines support for structured output generation^39,40^.

### Inference parameters and reproducibility

Given the logical reasoning nature of complication detection, we initially considered conservative parameters (temperature = 0). However, empirical evaluation revealed critical issues: smaller models frequently exhibited degeneration behavior at temperature=0, producing repetitive character outputs that rendered them non-functional for complex reasoning tasks. Additionally, even with deterministic settings and fixed seeds, model outputs exhibited inherent randomness across inference runs due to internal computational processes, floating-point precision variations, and parallel processing implementations.

Consequently, we adopted balanced parameters (temperature=0.6, top_p = 0.95) to address these challenges. This temperature setting strikes an appropriate balance between maintaining model functionality (preventing degeneration in smaller models) and preserving the reasoning consistency required for diagnostic decision-making. Our choice is further supported by recent research demonstrating that temperature changes from 0.0 to 1.0 do not have statistically significant impact on LLM problem-solving performance^41^. To ensure fair comparison across all models, we applied consistent temperature settings to eliminate temperature as a confounding variable.

To further mitigate the randomness introduced by temperature settings and ensure methodological consistency across all evaluations, we standardized our evaluation protocol to include five repeated inferences per case for all models, both commercial and open-source. This approach enables comprehensive reliability assessment and robust statistical analysis.

### Human expert annotation and gold standard development

Human expert evaluation utilized a rigorous, blinded assessment protocol. Identical prompts to those provided to the LLMs were presented to three experienced attending physicians specializing in perioperative medicine (all with >5 years of clinical experience). To assess inter-rater reliability, a randomly selected subset of 30 cases was independently annotated by all three experts, who were blinded to each other’s annotations. Fleiss’ κ statistic was calculated for all complication categories combined, yielding κ = 0.903 (95% CI: 0.839-0.952), indicating almost perfect agreement according to Landis and Koch criteria^42^. Cases were randomly distributed among these three experts to ensure unbiased evaluation. Additionally, each expert independently conducted comprehensive documentation quality assessment using the Patient Document Quality Index-9 (PDQI-9) scoring system^43^. The PDQI-9 evaluation encompassed nine critical dimensions of clinical documentation quality: completeness of patient history, clarity of clinical presentation, adequacy of examination findings, laboratory data integration, imaging report comprehensiveness, treatment plan documentation, medication reconciliation accuracy, discharge planning completeness, and overall narrative coherence. Each dimension was scored on a standardized scale, with final PDQI-9 scores calculated as the mean across all nine components. Inter-rater reliability for PDQI-9 assessments was evaluated using intraclass correlation coefficients, ensuring consistent quality evaluation across the expert panel.

The development of our gold standard incorporated both human expert opinions and initial LLM predictions as comprehensive reference points. All diagnostic outputs underwent systematic review by the three-physician panel. Final gold standard determinations necessitated a majority consensus (≥ 2 physicians). This gold standard served dual purposes: model evaluation and training data for fine-tuning. For fine-tuning of reasoning models, specifically QwQ 32B, all experts meticulously reviewed and refined the model’s reasoning outputs. The revised reasoning subsequently served as ‘golden reasoning’ templates, enclosed within tags, during the fine-tuning process.

#### Evaluation metrics

We employed standard multi-class classification metrics including F1-score, precision, and recall, calculated using both micro-averaged and macro-averaged approaches. F1-score served as the primary performance indicator due to its balanced consideration of both precision and recall, particularly important in clinical contexts.

Micro-averaged metrics were calculated by aggregating true positives, false positives, and false negatives across all cases globally before computing final metrics. Macro-averaged metrics were computed by calculating precision, recall, and F1-score for each individual case and then averaging these scores across all cases.

Each clinical case is represented as a set of complications detected. For a given case $i$:

- ${Y}_{i}$ = Ground truth label set (gold standard)

- ${\hat{Y}}_{i}$ = Predicted label set from model

- $n$ = Total number of cases in the evaluation dataset

From model outputs containing structured JSON or text responses, we extract complication labels using pattern matching and parsing algorithms. Each extracted label represents a specific perioperative complication according to the European Perioperative Clinical Outcome (EPCO) definitions.

For each case $i$, we calculate:

1 $${\text{True Positives}}\,({TP}):\,\,\,\,\,\,\,\,\,{\rm{T}}{{\rm{P}}}_{{\rm{i}}}=\left|{{\rm{Y}}}_{{\rm{i}}}\cap {\hat{{\rm{Y}}}}_{{\rm{i}}}\right|$$

2 $${\text{False Positives}}\,({\text{FP}}):\,\,\,\,\,\,\,\,\,{\rm{F}}{{\rm{P}}}_{{\rm{i}}}=\left|{\hat{{\rm{Y}}}}_{{\rm{i}}}-{{\rm{Y}}}_{{\rm{i}}}\right|$$

3 $${\text{False Negatives}}\,({\text{FN}}):\,\,\,\,\,\,\,{\rm{F}}{{\rm{N}}}_{{\rm{i}}}=\left|{{\rm{Y}}}_{{\rm{i}}}-{\hat{{\rm{Y}}}}_{{\rm{i}}}\right|$$

Where: - $\left|S\right|$ denotes the cardinality (size) of set $S$ - $\cap$ denotes set intersection - $-$ denotes set difference - ${Y}_{i}\cap {\hat{Y}}_{i}$ represents correctly predicted complications for case $i$ - ${\hat{Y}}_{i}-{Y}_{i}$ represents incorrectly predicted complications (model predicted but not in ground truth) - ${Y}_{i}-{\hat{Y}}_{i}$ represents missed complications (in ground truth but not predicted by model)

Micro-averaging aggregates the contributions of all cases to compute global metrics:

a. Global Aggregation:

4 $$T{P}_{{global}}=\mathop{\sum }\limits_{i=1}^{n}T{P}_{i}=\mathop{\sum }\limits_{i=1}^{n}\left|{Y}_{i}\cap {\hat{Y}}_{i}\right|$$

5 $$F{P}_{{global}}=\mathop{\sum }\limits_{i=1}^{n}F{P}_{i}=\mathop{\sum }\limits_{i=1}^{n}\left|{\hat{Y}}_{i}-{Y}_{i}\right|$$

6 $$F{N}_{{global}}=\mathop{\sum }\limits_{i=1}^{n}F{N}_{i}=\mathop{\sum }\limits_{i=1}^{n}\left|{Y}_{i}-{\hat{Y}}_{i}\right|$$

b. Micro-Averaged Precision:

7 $${Precisio}{n}_{{micro}}=\frac{T{P}_{{global}}}{T{P}_{{global}}+F{P}_{{global}}}$$

Where:

- $T{P}_{{global}}$ = Total correctly predicted complications across all cases
- $F{P}_{{global}}$ = Total incorrectly predicted complications across all cases
- $T{P}_{{global}}+F{P}_{{global}}$ = Total predicted complications across all cases

c. Micro-Averaged Recall:

8 $${Recal}{l}_{{micro}}=\frac{T{P}_{{global}}}{T{P}_{{global}}+F{N}_{{global}}}$$

Where:

- $F{N}_{{global}}$ = Total missed complications across all cases
- $T{P}_{{global}}+F{N}_{{global}}$ = Total actual complications across all cases

d. Micro-Averaged F1-Score:

9 $$F{1}_{{micro}}=\frac{2\times {Precisio}{n}_{{micro}}\times {Recal}{l}_{{micro}}}{{Precisio}{n}_{{micro}}+{Recal}{l}_{{micro}}}$$

Edge Case Handling

- If $$T{P}_{{global}}+F{P}_{{global}}=0$$: $${Precisio}{n}_{{micro}}=0.0$$
- If $$T{P}_{{global}}+F{N}_{{global}}=0$$: $${Recal}{l}_{{micro}}=0.0$$
- If $${Precisio}{n}_{{micro}}+{Recal}{l}_{{micro}}=0$$: $$F{1}_{{micro}}=0.0$$

For macro-averaging, we computes metrics for each case individually, then averages across all cases:

a. Per-Case Metrics: For each case $i$:

10 $${Precisio}{n}_{i}=\frac{T{P}_{i}}{T{P}_{i}+F{P}_{i}}=\frac{\left|{Y}_{i}\cap {\hat{Y}}_{i}\right|}{\left|{\hat{Y}}_{i}\right|}$$

11 $${Recal}{l}_{i}=\frac{T{P}_{i}}{T{P}_{i}+F{N}_{i}}=\frac{\left|{Y}_{i}\cap {\hat{Y}}_{i}\right|}{\left|{Y}_{i}\right|}$$

12 $$F{1}_{i}=\frac{2\times {Precisio}{n}_{i}\times {Recal}{l}_{i}}{{Precisio}{n}_{i}+{Recal}{l}_{i}}$$

b. Macro-Averaged Metrics:

13 $${Precisio}{n}_{{macro}}=\frac{1}{n}\mathop{\sum }\limits_{i=1}^{n}P{recisio}{n}_{i}$$

14 $${Recal}{l}_{{macro}}=\frac{1}{n}\mathop{\sum }\limits_{i=1}^{n}R{ecal}{l}_{i}$$

15 $$F{1}_{{macro}}=\frac{1}{n}\mathop{\sum }\limits_{i=1}^{n}F{1}_{i}$$

Edge Case Handling for Individual Cases

- *If* $T{P}_{i}+F{P}_{i}=0$ *(no predictions for case* $i$*):* ${Precisio}{n}_{i}=0.0$
- *If* $T{P}_{i}+F{N}_{i}=0$ *(no ground truth labels for case* $i$*):* ${Recal}{l}_{i}=0.0$
- *If* $${Precisio}{n}_{i}+{Recal}{l}_{i}=0$$*:* $$F{1}_{i}=0.0$$

### Statistical analysis

To address fundamental methodological limitations in our original statistical approach, we implemented a comprehensive bootstrap methodology following established practices for paired medical AI comparisons. All evaluations employed standardized protocols with five independent inference runs per model (including commercial models) to ensure reproducibility and robust confidence interval estimation.

Statistical significance testing was performed using patient-level bootstrap resampling with 2000 bootstrap iterations. For each bootstrap iteration, patients were resampled with replacement from the dataset, and one random inference run was selected for each model strategy to account for both patient-level and inference-level variability.

For visual presentation in figures, confidence intervals for performance metrics were calculated using t-distribution methods based on the mean and standard error across five repeated inference runs, providing parametric estimates of measurement uncertainty. For formal statistical comparisons requiring hypothesis testing (typically reported in tables), we implemented patient-level bootstrap paired testing with single-sided tests to evaluate our sequential improvement hypothesis (baseline → Chain-of-Thought → Targeted strategy → Targeted+LoRA). Bootstrap resampling with 2000 iterations was applied to estimate robust confidence intervals for mean differences without distributional assumptions, ensuring valid statistical inference regardless of underlying data distributions.

Effect sizes were quantified using mean differences in performance metrics between strategies, emphasizing clinical significance alongside statistical significance. Multiple comparison correction was applied using Bonferroni adjustment to control family-wise error rate. All statistical comparisons report: (1) mean differences in performance metrics, (2) 95% bootstrap confidence intervals for differences, (3) *p*-values from patient-level bootstrap tests, and (4) corrected *p*-values after multiple comparison adjustment.

All data preprocessing, model evaluation, and statistical analyses were conducted using Python within Jupyter notebook environments. The complete codebase is slated for open-source release (https://github.com/gscfwid/PeriComp), accompanied by representative data examples. The full dataset will be accessible to qualified researchers upon reasonable request and with appropriate institutional review board approval.

## Supplementary information

Supplementary Information

## Data Availability

Clinical datasets are not publicly available due to patient privacy but can be requested from corresponding authors with appropriate institutional approval. Fine-tuned models are available at https://huggingface.co/collections/gscfwid/pericomp. Source code for analyses in this paper is publicly available at https://github.com/gscfwid/PeriComp.
