# Supplementary material for: Enhancing privacy-preserving deployable large language models for perioperative complication detection: a targeted strategy with LoRA fine-tuning
Source: NPJ Digit Med. 2025 Dec 13;8:773. doi: 10.1038/s41746-025-02139-3 (PMC12717251; doi:10.1038/s41746-025-02139-3)
Supplement: Supplementary file 1 — Supplementary Information [file 41746_2025_2139_MOESM1_ESM.pdf]

Supplementary Information

SUPPLEMENTARY FIGURES 2

SUPPLEMENTARY TABLES 10

AN EXAMPLE OF PROMPT (TRANSLATED TO ENGLISH) 29

Comprehensive original version *29*

Comprehensive modified version *38*

Targeted version（Taking acute kidney injury as an example） *44*

Supplementary Figures

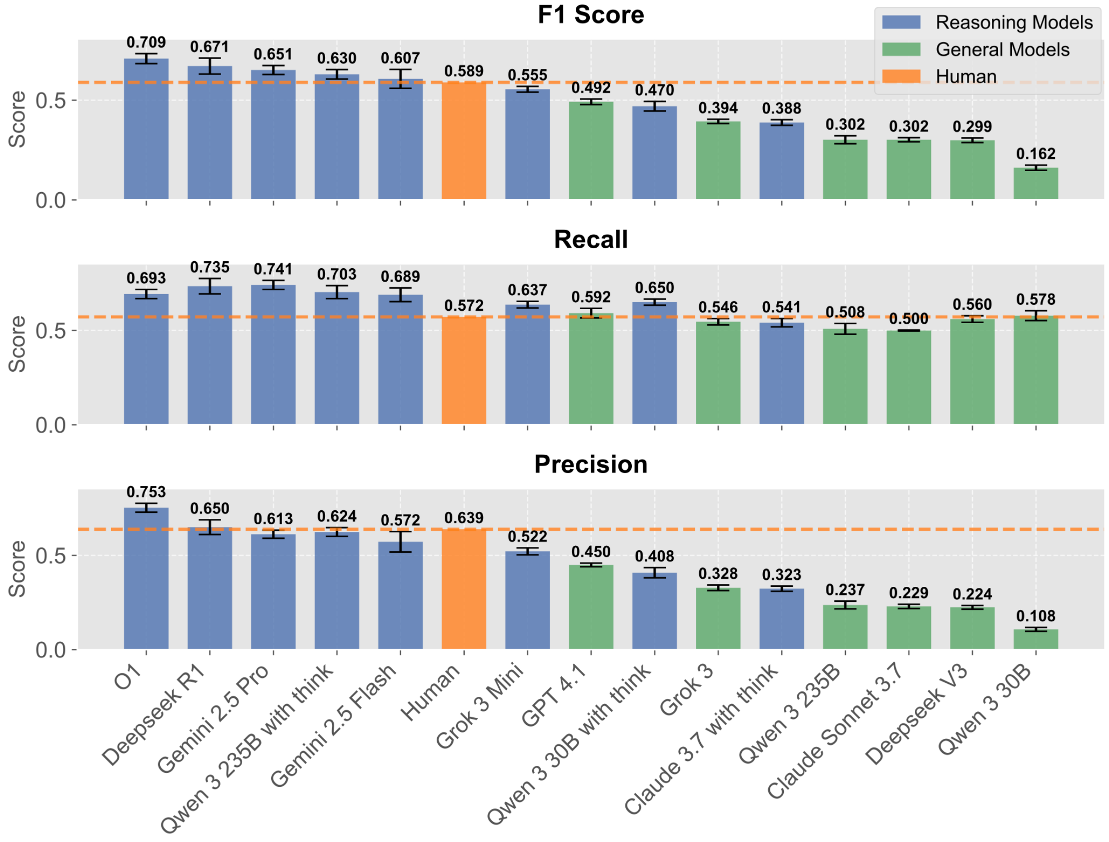

Supplementary Figure 1. Initial model performance evaluation using macro-averaged metrics. Performance evaluation across multiple state-of-the-art language models using macro-averaged metrics with confidence intervals from five repeated inferences, demonstrating superior performance of reasoning models over general models, with several AI models exceeding human expert benchmarks.

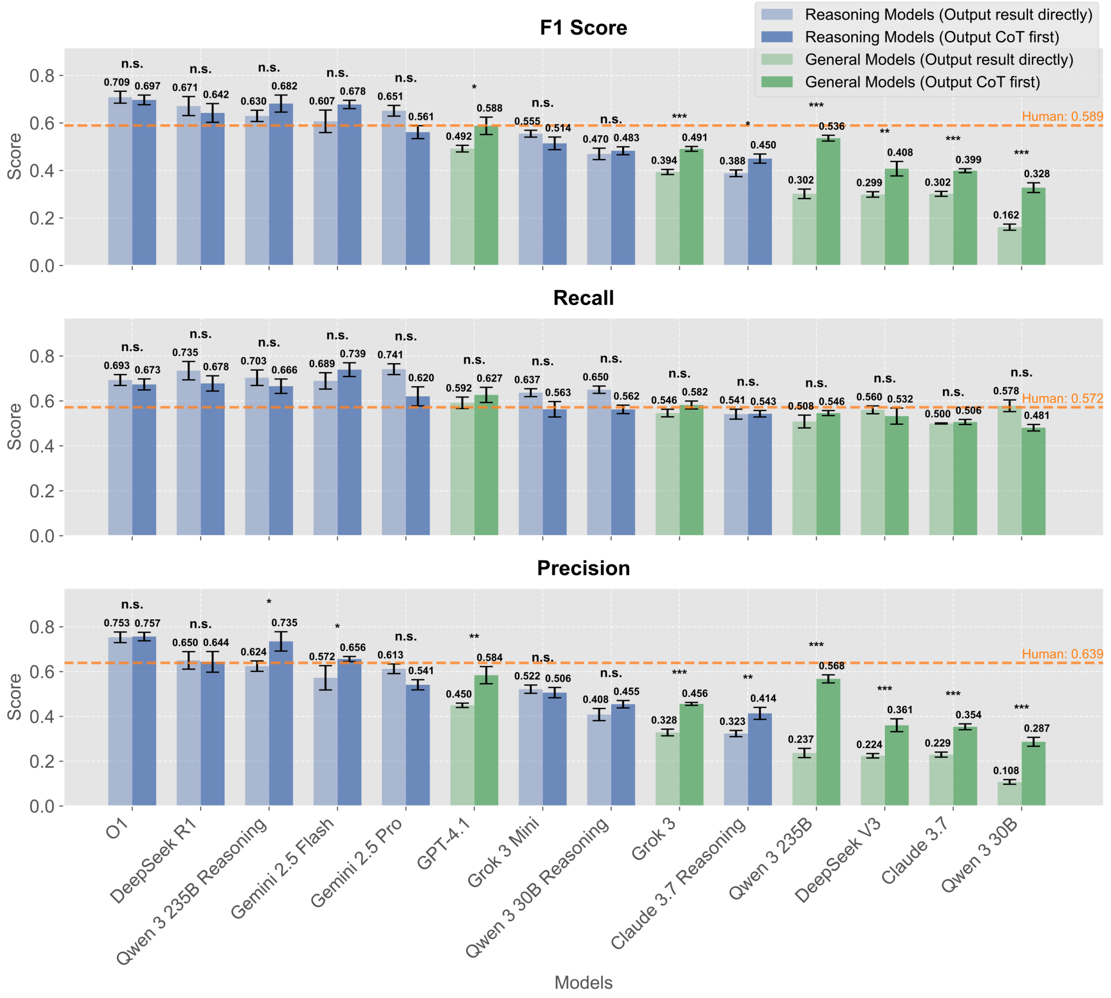

Supplementary Figure 2. Chain-of-Thought prompting performance comparison using macro-averaged metrics. Performance comparison following CoT implementation using macro-averaged metrics with 95% confidence intervals from five repeated inferences and patient-level bootstrap paired testing. Asterisks indicate statistical significance levels from bootstrap paired testing: *$p < 0.05$, **$p < 0.01$, ***$p < 0.001$, showing significant improvements in general models while reasoning models maintained consistently high performance across F1 score, recall, and precision metrics.

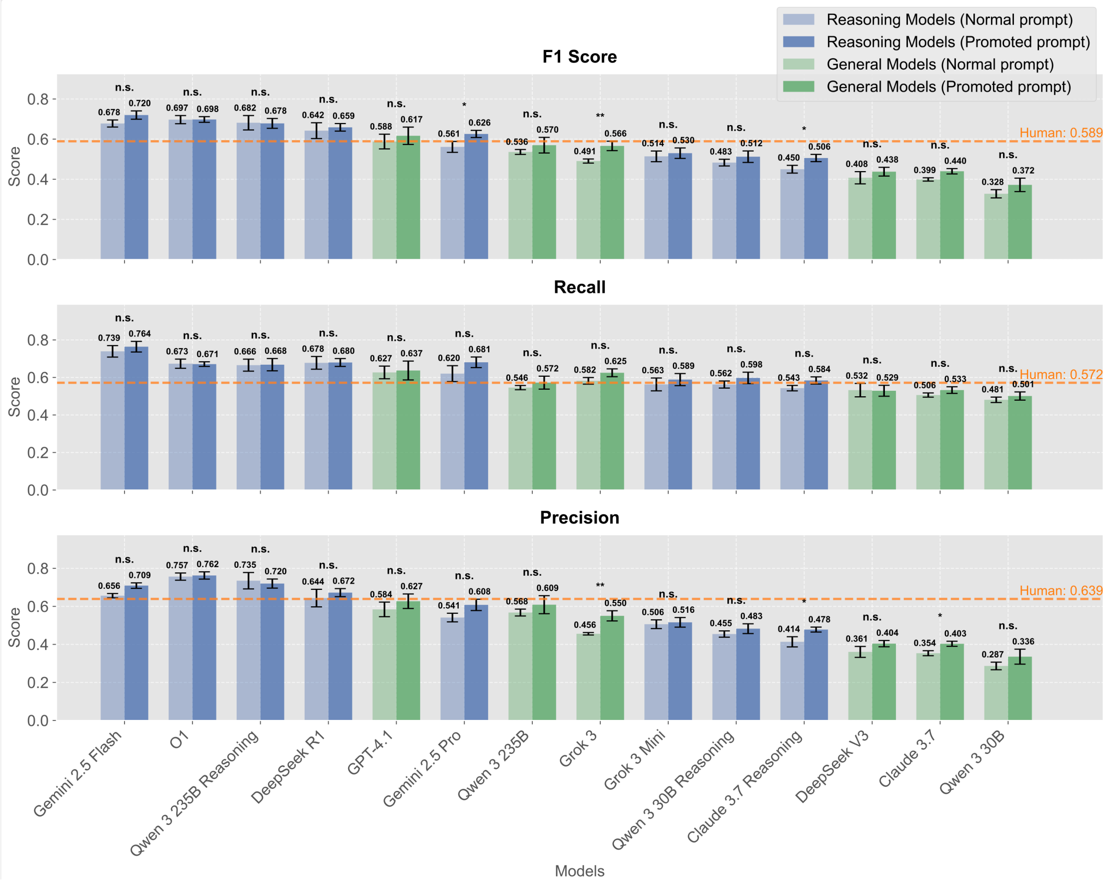

Supplementary Figure 3. Prompt optimization performance comparison using macro-averaged metrics. Performance comparison following CoT implementation using macro-averaged metrics with 95% confidence intervals from five repeated inferences and patient-level bootstrap paired testing. Asterisks indicate statistical significance levels from bootstrap paired testing: *$p < 0.05$, **$p < 0.01$, ***$p < 0.001$, showing significant improvements in general models while reasoning models maintained consistently high performance across F1 score, recall, and precision metrics.

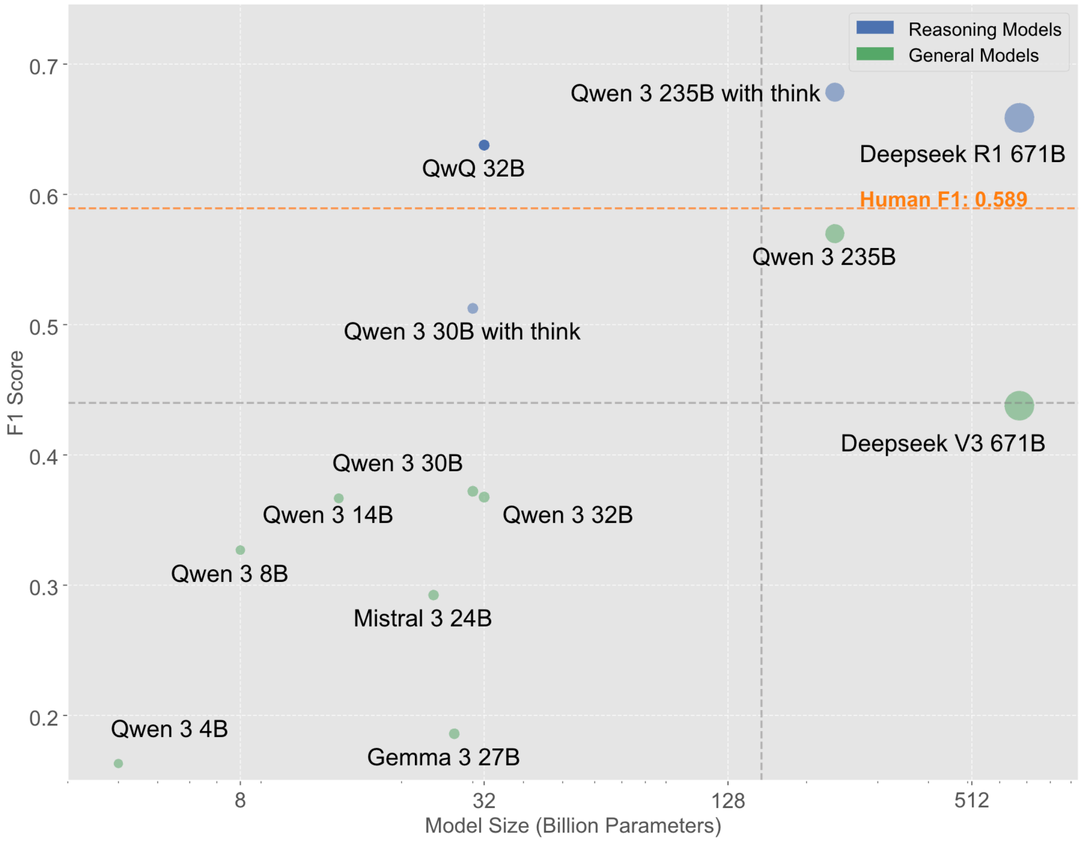

Supplementary Figure 4. Open-source model performance quadrant analysis using macro-averaged metrics. Systematic evaluation of open-source models spanning 4B to 671B parameters using macro-averaged metrics. Point sizes are proportional to model parameter counts. The quadrant chart demonstrates correlation between model size and performance.

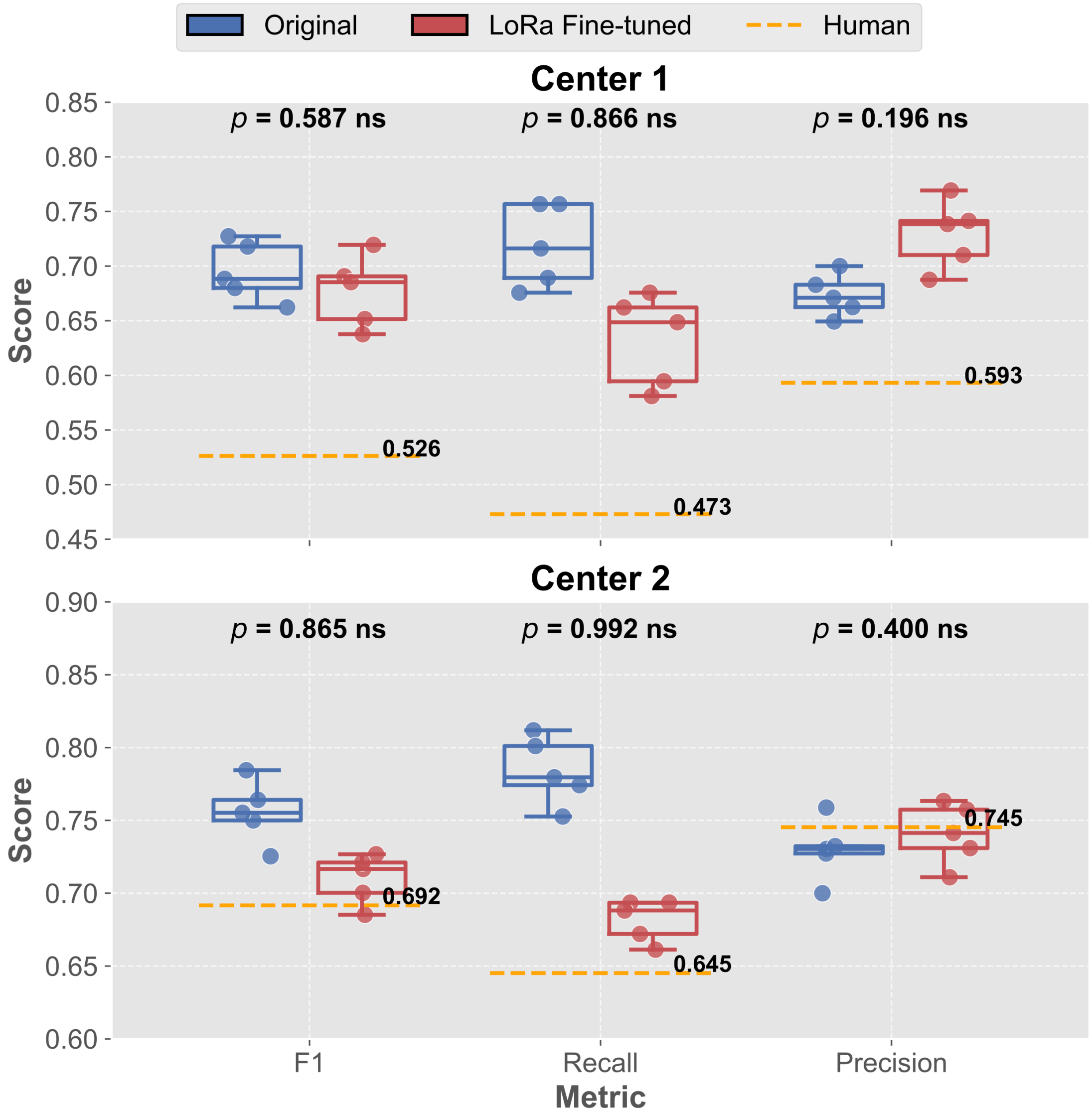

Supplementary Figure 5. QwQ 32B LoRa fine-tuning performance evaluation using macro-averaged metrics. Performance comparison before (blue) and after (red) LoRa fine-tuning across F1 score, recall, and precision metrics for both validation datasets using macro-averaged metrics and 95% confidence intervals from five repeated inferences with patient-level bootstrap paired testing. Box plots show distribution of results with no statistically significant improvements observed (all $p > 0.05$). Orange dashed lines represent human expert benchmarks for each metric in respective centers.

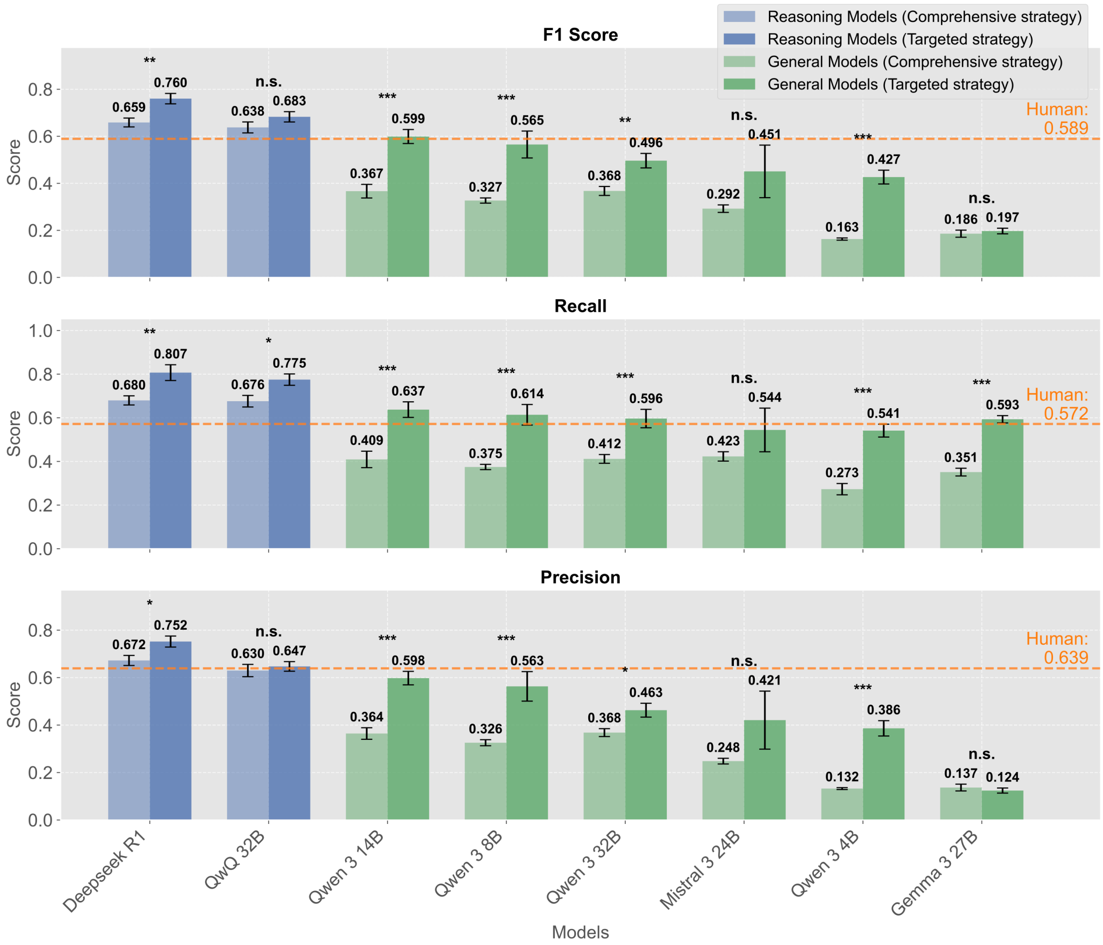

Supplementary Figure 6. Comprehensive versus targeted strategy comparison using macro-averaged metrics. Performance comparison between comprehensive strategy (lighter colors) and targeted strategy (darker colors) across F1 score, recall, and precision metrics using macro-averaged results with patient-level bootstrap paired testing. Reasoning models (light blue and blue) and general models (light green and green) demonstrate differential responses to strategy transition, with smaller models showing substantial and statistically significant improvements (Qwen3 4B-32B: all $p < 0.001$) while larger reasoning models maintain consistent performance. Orange dashed lines indicate human expert benchmarks for each metric.

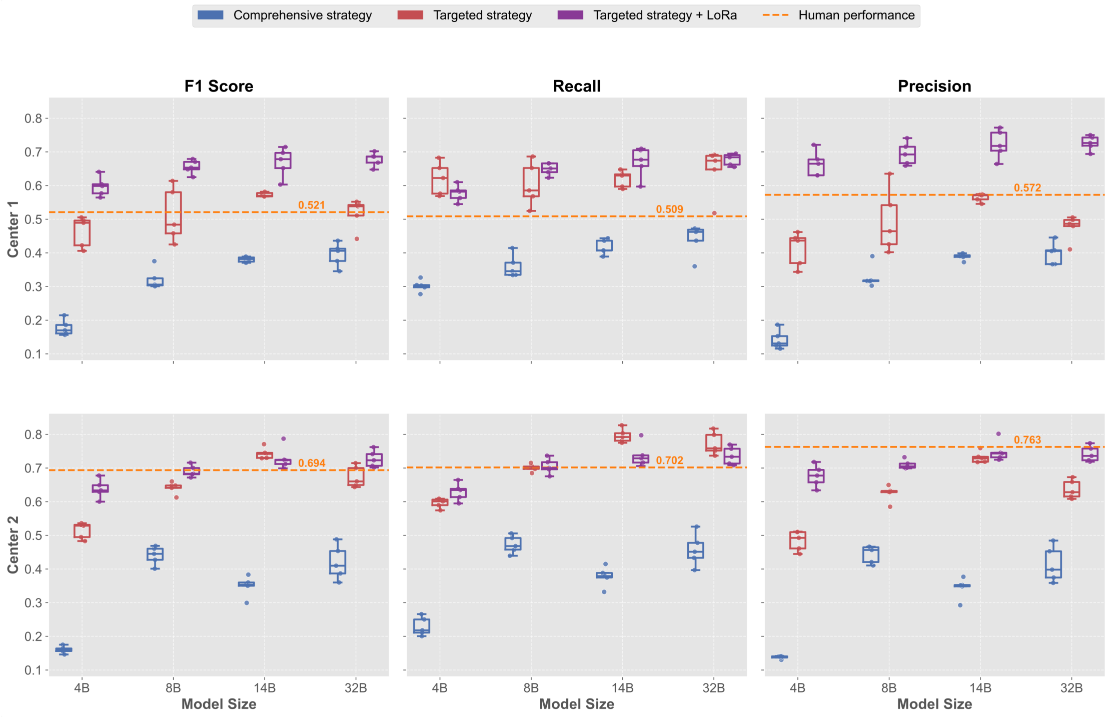

Supplementary Figure 7. LoRa fine-tuning optimization results for Qwen 3 models using macro-averaged metrics. Comprehensive performance comparison across F1 score, recall, and precision metrics for both Center 1 and Center 2 validation datasets using macro-averaged metrics with 95% confidence intervals from five repeated inferences and patient-level bootstrap paired testing. Box plots illustrate performance distributions for comprehensive strategy (blue), targeted strategy (red), and targeted strategy with LoRa fine-tuning (purple), compared against human expert benchmarks (orange dashed lines). Results demonstrate that LoRa fine-tuning significantly enhances smaller models' performance, with 4B and 8B parameter models showing substantial and statistically significant improvements.

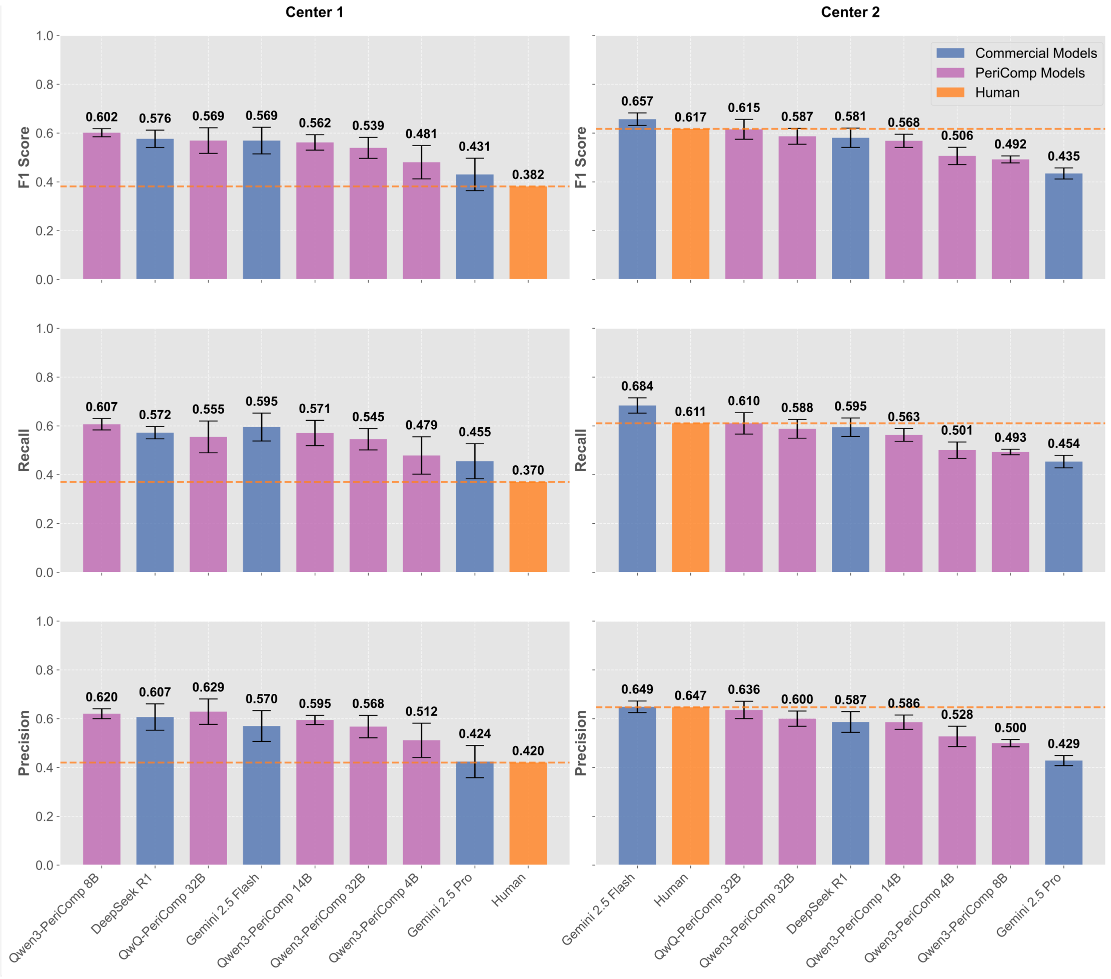

Supplementary Figure 8. Strict performance evaluation with PeriComp models using macro-averaged metrics. Comprehensive performance comparison across F1 score, recall, and precision metrics for both Center 1 and Center 2 validation datasets using macro-averaged metrics with 95% confidence intervals from five repeated inference. Evaluation under strict criteria requires correct identification of both specific complication type and severity grade (mild, moderate, or severe) for a diagnosis to be considered valid. Commercial models (blue) including Gemini 2.5 Flash, DeepSeek R1, and Gemini 2.5 Pro are compared against fine-tuned PeriComp models (purple) including QwQ-PeriComp-4B, QwQ-PeriComp-8B, QwQ-PeriComp-14B, and QwQ-PeriComp-32B, with human expert performance (orange) serving as clinical benchmarks. Orange dashed lines indicate human expert performance thresholds for each metric in respective centers.

Supplementary Tables

Supplementary Table 1: Statistical Analysis of Chain-of-Thought (CoT) Prompting Effects (Output CoT first vs Output result directly) with Bootstrap Paired Testing (Micro-average)

Model	Metric	Mean Diff	95% CI	p-value
**Claude 3.7 Sonnet**	F1	0.109	[0.066, 0.152]	**<0.001**
	Precision	0.129	[0.090, 0.173]	**<0.001**
	Recall	-0.001	[-0.063, 0.061]	0.554
**Claude 3.7 Reasoning**	F1	0.087	[0.037, 0.133]	**<0.001**
	Precision	0.117	[0.059, 0.171]	**<0.001**
	Recall	-0.004	[-0.068, 0.054]	0.568
**Qwen3 30B**	F1	0.141	[0.096, 0.196]	**<0.001**
	Precision	0.114	[0.077, 0.161]	**<0.001**
	Recall	-0.135	[-0.209, -0.056]	1.000
**Qwen3 235B**	F1	0.209	[0.142, 0.276]	**<0.001**
	Precision	0.308	[0.236, 0.380]	**<0.001**
	Recall	-0.049	[-0.138, 0.044]	0.871
**Qwen3 Reasoning 30B**	F1	0.066	[-0.020, 0.149]	0.067
	Precision	0.114	[0.029, 0.195]	**0.003**
	Recall	-0.104	[-0.177, -0.037]	0.999

Model	Metric	Mean Diff	95% CI	p-value
**Qwen3 Reasoning 235B**	F1	0.115	[0.026, 0.216]	**0.006**
	Precision	0.247	[0.116, 0.382]	**<0.001**
	Recall	-0.056	[-0.137, 0.033]	0.883
**Gemini 2.5 Flash**	F1	0.057	[-0.009, 0.132]	0.051
	Precision	0.073	[-0.001, 0.158]	**0.028**
	Recall	0.029	[-0.039, 0.104]	0.239
**Gemini 2.5 Pro**	F1	-0.019	[-0.088, 0.040]	0.711
	Precision	0.031	[-0.085, 0.108]	0.230
	Recall	-0.088	[-0.162, -0.016]	0.994
**OpenAI o1**	F1	-0.014	[-0.070, 0.037]	0.683
	Precision	0.008	[-0.047, 0.057]	0.381
	Recall	-0.029	[-0.091, 0.030]	0.834
**GPT-4o**	F1	0.050	[-0.032, 0.147]	0.128
	Precision	0.087	[-0.010, 0.199]	**0.039**
	Recall	-0.013	[-0.094, 0.068]	0.642
**Grok-3**	F1	0.089	[0.047, 0.129]	**<0.001**
	Precision	0.122	[0.082, 0.164]	**<0.001**
	Recall	0.002	[-0.053, 0.057]	0.508
**Grok-3 Mini**	F1	-0.028	[-0.090, 0.037]	0.806
	Precision	0.001	[-0.063, 0.070]	0.495

Model	Metric	Mean Diff	95% CI	p-value
	Recall	-0.073	[-0.147, -0.004]	0.982
**DeepSeek R1**	F1	-0.019	[-0.086, 0.054]	0.708
	Precision	0.020	[-0.055, 0.101]	0.321
	Recall	-0.067	[-0.135, 0.005]	0.968
**DeepSeek V3**	F1	0.155	[0.090, 0.216]	**<0.001**
	Precision	0.171	[0.112, 0.224]	**<0.001**
	Recall	-0.055	[-0.132, 0.022]	0.920

Statistical Methods: Patient-level bootstrap paired testing with 2,000 iterations. All tests are single-sided (greater than).

Abbreviations: CoT = Chain-of-Thought; CI = Confidence Interval; Mean Diff = Mean Difference

Note: Positive values indicate improvement when adding Chain-of-Thought prompting.

Supplementary Table 2: Statistical Analysis of Chain-of-Thought (CoT) Prompting Effects (Output CoT first vs Output result directly) with Bootstrap Paired Testing (Macro-average)

Model	Metric	Mean Diff	95% CI	p-value
**Claude 3.7 Sonnet**	F1	0.096	[0.049, 0.142]	**<0.001**
	Precision	0.123	[0.076, 0.171]	**<0.001**
	Recall	0.006	[-0.050, 0.064]	0.413

Model	Metric	Mean Diff	95% CI	p-value
**Claude 3.7 Reasoning**	F1	0.061	[0.005, 0.120]	**0.017**
	Precision	0.089	[0.029, 0.156]	**0.001**
	Recall	0.001	[-0.065, 0.067]	0.493
**Qwen3 30B**	F1	0.167	[0.110, 0.229]	**<0.001**
	Precision	0.180	[0.125, 0.243]	**<0.001**
	Recall	-0.095	[-0.182, -0.008]	0.983
**Qwen3 235B**	F1	0.235	[0.156, 0.311]	**<0.001**
	Precision	0.331	[0.248, 0.408]	**<0.001**
	Recall	0.039	[-0.066, 0.141]	0.232
**Qwen3 Reasoning 30B**	F1	0.012	[-0.065, 0.094]	0.388
	Precision	0.045	[-0.036, 0.127]	0.147
	Recall	-0.089	[-0.167, -0.006]	0.984
**Qwen3 Reasoning 235B**	F1	0.052	[-0.039, 0.149]	0.147
	Precision	0.111	[0.015, 0.219]	**0.012**
	Recall	-0.037	[-0.138, 0.062]	0.762
**Gemini 2.5 Flash**	F1	0.071	[-0.013, 0.167]	0.054
	Precision	0.084	[-0.004, 0.188]	**0.033**
	Recall	0.051	[-0.035, 0.147]	0.138
**Gemini 2.5 Pro**	F1	-0.090	[-0.178, -0.015]	0.990
	Precision	-0.071	[-0.158, 0.006]	0.966

Model	Metric	Mean Diff	95% CI	p-value
	Recall	-0.120	[-0.214, -0.021]	0.990
**OpenAI o1**	F1	-0.012	[-0.071, 0.057]	0.654
	Precision	0.003	[-0.057, 0.066]	0.488
	Recall	-0.021	[-0.085, 0.045]	0.734
**GPT-4o**	F1	0.098	[0.010, 0.194]	**0.014**
	Precision	0.136	[0.050, 0.229]	**0.001**
	Recall	0.037	[-0.063, 0.141]	0.227
**Grok-3**	F1	0.097	[0.050, 0.147]	**<0.001**
	Precision	0.128	[0.081, 0.178]	**<0.001**
	Recall	0.036	[-0.024, 0.096]	0.123
**Grok-3 Mini**	F1	-0.040	[-0.118, 0.030]	0.858
	Precision	-0.015	[-0.095, 0.060]	0.650
	Recall	-0.073	[-0.167, 0.004]	0.966
**DeepSeek R1**	F1	-0.030	[-0.130, 0.071]	0.710
	Precision	-0.007	[-0.113, 0.100]	0.555
	Recall	-0.058	[-0.157, 0.038]	0.871
**DeepSeek V3**	F1	0.107	[0.041, 0.172]	**0.001**
	Precision	0.135	[0.072, 0.197]	**<0.001**
	Recall	-0.031	[-0.121, 0.053]	0.758

Statistical Methods: Patient-level bootstrap paired testing with 2,000 iterations. All tests are single-sided (greater than).

Abbreviations: CoT = Chain-of-Thought; CI = Confidence Interval; Mean Diff = Mean Difference

Note: Positive values indicate improvement when adding Chain-of-Thought prompting.

Supplementary Table 3: Statistical Analysis of Prompt Optimization Effects (Promote vs Normal prompts) with Bootstrap Paired Testing (Micro-average)

Model	Metric	Mean Diff	95% CI	p-value
**Claude 3.7 Sonnet**	F1	0.035	[-0.020, 0.086]	0.088
	Precision	0.042	[-0.011, 0.091]	0.058
	Recall	0.016	[-0.050, 0.082]	0.319
**Claude 3.7 Reasoning**	F1	0.041	[-0.018, 0.100]	0.086
	Precision	0.053	[-0.011, 0.120]	**0.049**
	Recall	0.018	[-0.050, 0.087]	0.324
**Qwen3 30B**	F1	0.050	[-0.020, 0.114]	0.087
	Precision	0.047	[-0.013, 0.105]	0.071
	Recall	0.010	[-0.062, 0.086]	0.420
**Qwen3 235B**	F1	0.038	[-0.042, 0.109]	0.178
	Precision	0.049	[-0.051, 0.141]	0.169
	Recall	0.027	[-0.048, 0.103]	0.259
**Qwen3 Reasoning 30B**	F1	0.046	[-0.014, 0.102]	0.065
	Precision	0.048	[-0.007, 0.099]	**0.045**

Model	Metric	Mean Diff	95% CI	p-value
	Recall	0.038	[-0.037, 0.109]	0.179
**Qwen3 Reasoning 235B**	F1	0.007	[-0.074, 0.087]	0.427
	Precision	0.001	[-0.104, 0.096]	0.474
	Recall	0.011	[-0.072, 0.096]	0.414
**Gemini 2.5 Flash**	F1	0.030	[-0.034, 0.092]	0.166
	Precision	0.044	[-0.029, 0.114]	0.104
	Recall	0.009	[-0.072, 0.088]	0.432
**Gemini 2.5 Pro**	F1	0.035	[-0.043, 0.108]	0.164
	Precision	0.031	[-0.111, 0.165]	0.246
	Recall	0.040	[-0.039, 0.120]	0.182
**OpenAI o1**	F1	-0.008	[-0.055, 0.039]	0.619
	Precision	0.006	[-0.042, 0.058]	0.416
	Recall	-0.016	[-0.069, 0.033]	0.761
**GPT-4o**	F1	0.041	[-0.078, 0.141]	0.228
	Precision	0.070	[-0.066, 0.181]	0.151
	Recall	0.005	[-0.105, 0.097]	0.451
**Grok-3**	F1	0.054	[0.005, 0.105]	**0.015**
	Precision	0.079	[0.030, 0.133]	**0.002**
	Recall	0.015	[-0.044, 0.074]	0.342
**Grok-3 Mini**	F1	0.000	[-0.065, 0.065]	0.506

Model	Metric	Mean Diff	95% CI	p-value
	Precision	-0.010	[-0.078, 0.055]	0.602
	Recall	0.014	[-0.056, 0.088]	0.371
**DeepSeek R1**	F1	0.021	[-0.049, 0.085]	0.268
	Precision	0.039	[-0.037, 0.109]	0.153
	Recall	0.001	[-0.071, 0.073]	0.498
**DeepSeek V3**	F1	0.045	[-0.015, 0.110]	0.081
	Precision	0.061	[0.011, 0.116]	**0.011**
	Recall	0.001	[-0.079, 0.088]	0.511

Statistical Methods: Patient-level bootstrap paired testing with 2,000 iterations. All tests are single-sided (greater than).

Abbreviations: CI = Confidence Interval; Mean Diff = Mean Difference

Note: Positive values indicate improvement with prompt optimization.

Supplementary Table 4: Statistical Analysis of Prompt Optimization Effects (Promote vs Normal prompts) with Bootstrap Paired Testing (Macro-average)

Model	Metric	Mean Diff	95% CI	p-value
**Claude 3.7 Sonnet**	F1	0.041	[-0.015, 0.097]	0.068
	Precision	0.050	[-0.008, 0.107]	**0.046**
	Recall	0.026	[-0.038, 0.091]	0.219

Model	Metric	Mean Diff	95% CI	p-value
**Claude 3.7 Reasoning**	F1	0.055	[-0.009, 0.122]	**0.049**
	Precision	0.064	[-0.005, 0.133]	**0.037**
	Recall	0.041	[-0.028, 0.110]	0.123
**Qwen3 30B**	F1	0.045	[-0.035, 0.117]	0.133
	Precision	0.050	[-0.036, 0.127]	0.130
	Recall	0.021	[-0.055, 0.095]	0.294
**Qwen3 235B**	F1	0.032	[-0.063, 0.118]	0.252
	Precision	0.038	[-0.065, 0.133]	0.240
	Recall	0.025	[-0.064, 0.113]	0.298
**Qwen3 Reasoning 30B**	F1	0.028	[-0.049, 0.107]	0.235
	Precision	0.027	[-0.051, 0.102]	0.250
	Recall	0.033	[-0.053, 0.118]	0.226
**Qwen3 Reasoning 235B**	F1	-0.003	[-0.102, 0.088]	0.514
	Precision	-0.015	[-0.123, 0.081]	0.594
	Recall	0.004	[-0.097, 0.103]	0.464
**Gemini 2.5 Flash**	F1	0.042	[-0.030, 0.117]	0.129
	Precision	0.053	[-0.015, 0.122]	0.060
	Recall	0.026	[-0.060, 0.116]	0.290
**Gemini 2.5 Pro**	F1	0.066	[-0.009, 0.147]	**0.049**
	Precision	0.068	[-0.023, 0.154]	0.065

Model	Metric	Mean Diff	95% CI	p-value
	Recall	0.062	[-0.046, 0.153]	0.111
**OpenAI o1**	F1	0.001	[-0.058, 0.057]	0.490
	Precision	0.006	[-0.051, 0.068]	0.444
	Recall	-0.002	[-0.065, 0.055]	0.513
**GPT-4o**	F1	0.028	[-0.084, 0.117]	0.290
	Precision	0.042	[-0.066, 0.132]	0.216
	Recall	0.010	[-0.112, 0.109]	0.397
**Grok-3**	F1	0.074	[0.012, 0.145]	**0.009**
	Precision	0.093	[0.029, 0.164]	**0.002**
	Recall	0.042	[-0.030, 0.117]	0.129
**Grok-3 Mini**	F1	0.014	[-0.054, 0.084]	0.372
	Precision	0.008	[-0.061, 0.082]	0.434
	Recall	0.024	[-0.053, 0.116]	0.313
**DeepSeek R1**	F1	0.018	[-0.075, 0.111]	0.359
	Precision	0.030	[-0.072, 0.133]	0.288
	Recall	0.003	[-0.087, 0.095]	0.476
**DeepSeek V3**	F1	0.030	[-0.042, 0.109]	0.220
	Precision	0.043	[-0.025, 0.115]	0.116
	Recall	-0.005	[-0.089, 0.093]	0.547

Statistical Methods: Patient-level bootstrap paired testing with 2,000 iterations. All tests are single-sided (greater than).

Abbreviations: CI = Confidence Interval; Mean Diff = Mean Difference

Note: Positive values indicate improvement with prompt optimization.

Supplementary Table 5: Statistical Analysis of QwQ 32B LoRa Fine-tuning Effects (Micro-averaged) with Bootstrap Paired Testing

Center	Metric	Mean Diff	95% CI	p-value
**Center 1**	F1	-0.017	[-0.148, 0.118]	0.587
**(n=46)**	Precision	0.058	[-0.082, 0.196]	0.196
	Recall	-0.085	[-0.246, 0.072]	0.866
**Center 2**	F1	-0.046	[-0.125, 0.035]	0.865
**(n=102)**	Precision	0.011	[-0.070, 0.097]	0.400
	Recall	-0.102	[-0.191, -0.016]	0.992

Statistical Methods: Patient-level bootstrap paired testing with 2,000 iterations. All tests are single-sided (greater than).

Abbreviations: LoRa = Low-Rank Adaptation; CI = Confidence Interval; Mean Diff = Mean Difference

Supplementary Table 6: Statistical Analysis of QwQ 32B LoRa Fine-tuning Effects (Macro-averaged) with Bootstrap Paired Testing

Center	Metric	Mean Diff	95% CI	p-value
**Center 1**	F1	0.016	[-0.147, 0.177]	0.430

Center	Metric	Mean Diff	95% CI	p-value
**(n=46)**	Precision	0.096	[-0.065, 0.262]	0.123
	Recall	-0.033	[-0.217, 0.145]	0.640
**Center 2**	F1	-0.046	[-0.151, 0.060]	0.795
**(n=102)**	Precision	-0.016	[-0.119, 0.092]	0.622
	Recall	-0.080	[-0.184, 0.029]	0.926

Statistical Methods: Patient-level bootstrap paired testing with 2,000 iterations. All tests are single-sided (greater than).

Abbreviations: LoRa = Low-Rank Adaptation; CI = Confidence Interval; Mean Diff = Mean Difference

Supplementary Table 7: Statistical Analysis of Comprehensive vs Targeted Strategy Effects (Micro-averaged) with Bootstrap Paired Testing

Model	Metric	Mean Diff	95% CI	p-value
**DeepSeek R1**	F1	0.046	[-0.020, 0.113]	0.088
	Precision	-0.009	[-0.095, 0.089]	0.600
	Recall	0.112	[0.030, 0.190]	**0.003**
**QwQ 32B**	F1	0.008	[-0.057, 0.077]	0.417
	Precision	-0.046	[-0.114, 0.031]	0.899
	Recall	0.089	[0.009, 0.169]	**0.017**
**Qwen3 4B**	F1	0.227	[0.165, 0.293]	**<0.001**
	Precision	0.202	[0.147, 0.257]	**<0.001**

Model	Metric	Mean Diff	95% CI	p-value
	Recall	0.241	[0.148, 0.336]	**<0.001**
**Qwen3 8B**	F1	0.244	[0.128, 0.342]	**<0.001**
	Precision	0.239	[0.111, 0.354]	**<0.001**
	Recall	0.245	[0.139, 0.338]	**<0.001**
**Qwen3 14B**	F1	0.194	[0.118, 0.274]	**<0.001**
	Precision	0.183	[0.110, 0.261]	**<0.001**
	Recall	0.206	[0.106, 0.304]	**<0.001**
**Qwen3 32B**	F1	0.143	[0.074, 0.212]	**<0.001**
	Precision	0.116	[0.049, 0.179]	**<0.001**
	Recall	0.186	[0.093, 0.278]	**<0.001**
**Mistral 24B**	F1	0.157	[-0.015, 0.274]	0.052
	Precision	0.164	[-0.003, 0.272]	**0.035**
	Recall	0.103	[-0.061, 0.240]	0.123
**Gemma2 27B**	F1	0.011	[-0.028, 0.047]	0.279
	Precision	-0.011	[-0.040, 0.014]	0.782
	Recall	0.291	[0.211, 0.371]	**<0.001**

Statistical Methods: Patient-level bootstrap paired testing with 2,000 iterations. All tests are single-sided (greater than).

Abbreviations: CI = Confidence Interval; Mean Diff = Mean Difference

Note: Positive values indicate improvement with targeted strategy.

Supplementary Table 8: Statistical Analysis of Comprehensive vs Targeted Strategy Effects (Macro-averaged) with Bootstrap Paired Testing

Model	Metric	Mean Diff	95% CI	p-value
**DeepSeek R1**	F1	0.100	[0.018, 0.186]	**0.006**
	Precision	0.078	[-0.011, 0.172]	**0.042**
	Recall	0.127	[0.035, 0.220]	**0.002**
**QwQ 32B**	F1	0.043	[-0.045, 0.124]	0.162
	Precision	0.016	[-0.078, 0.100]	0.343
	Recall	0.097	[0.000, 0.188]	**0.026**
**Qwen3 4B**	F1	0.262	[0.187, 0.335]	**<0.001**
	Precision	0.252	[0.176, 0.327]	**<0.001**
	Recall	0.266	[0.169, 0.366]	**<0.001**
**Qwen3 8B**	F1	0.238	[0.115, 0.344]	**<0.001**
	Precision	0.237	[0.108, 0.350]	**<0.001**
	Recall	0.239	[0.118, 0.349]	**<0.001**
**Qwen3 14B**	F1	0.235	[0.132, 0.336]	**<0.001**
	Precision	0.236	[0.134, 0.335]	**<0.001**
	Recall	0.231	[0.114, 0.344]	**<0.001**
**Qwen3 32B**	F1	0.129	[0.033, 0.224]	**0.002**
	Precision	0.094	[-0.001, 0.189]	**0.026**
	Recall	0.186	[0.077, 0.298]	**<0.001**
**Mistral 24B**	F1	0.159	[-0.040, 0.283]	0.102

Model	Metric	Mean Diff	95% CI	p-value
	Precision	0.173	[-0.037, 0.302]	0.106
	Recall	0.122	[-0.072, 0.262]	0.126
**Gemma2 27B**	F1	0.012	[-0.030, 0.053]	0.287
	Precision	-0.012	[-0.048, 0.025]	0.763
	Recall	0.243	[0.171, 0.313]	**<0.001**

Statistical Methods: Patient-level bootstrap paired testing with 2,000 iterations. All tests are single-sided (greater than).

Abbreviations: CI = Confidence Interval; Mean Diff = Mean Difference

Note: Positive values indicate improvement with targeted strategy.

Supplementary Table 9: Statistical Analysis of Model Performance Improvements with Bootstrap Paired Testing (Macro-averaged)

Center	Model	Comparison	Metric	Mean Diff	95% CI	p-value	p-corrected
	**Qwen 4B**	Comprehensive vs Targeted	F1	0.285	[0.140, 0.423]	<0.001	**0.001***
			Precision	0.269	[0.117, 0.410]	<0.001	**0.001***
			Recall	0.316	[0.141, 0.496]	<0.001	**0.001***
**Center 1 (n=46)**		Targeted vs Targeted + SFT	F1	0.133	[-0.046, 0.308]	0.071	0.141
			Precision	0.254	[0.064, 0.443]	**0.004**	**0.007***
			Recall	-0.043	[-0.238, 0.151]	0.654	1.000
	**Qwen 8B**	Comprehensive vs Targeted	F1	0.189	[-0.001, 0.405]	**0.027**	**0.054**
			Precision	0.163	[-0.037, 0.411]	0.072	0.144

Center	Model	Comparison	Metric	Mean Diff	95% CI	p-value	p-corrected
			Recall	0.244	[0.047, 0.460]	**0.012**	**0.024**
		Targeted vs Targeted + SFT	F1	0.144	[-0.061, 0.340]	0.090	0.180
			Precision	0.201	[-0.030, 0.412]	**0.045**	0.089
			Recall	0.048	[-0.153, 0.250]	0.336	0.671
	**Qwen 14B**	Comprehensive vs Targeted	F1	0.193	[0.042, 0.347]	**0.005**	**0.010**
			Precision	0.177	[0.017, 0.348]	**0.015**	**0.029**
			Recall	0.203	[0.030, 0.371]	**0.012**	**0.023**
		Targeted vs Targeted + SFT	F1	0.096	[-0.072, 0.256]	0.131	0.261
			Precision	0.159	[-0.012, 0.324]	**0.035**	0.069
			Recall	0.049	[-0.130, 0.228]	0.309	0.617
	**Qwen 32B**	Comprehensive vs Targeted	F1	0.124	[-0.042, 0.287]	0.068	0.136
			Precision	0.080	[-0.084, 0.241]	0.174	0.348
			Recall	0.207	[-0.018, 0.415]	**0.040**	0.080
		Targeted vs Targeted + SFT	F1	0.162	[-0.004, 0.333]	**0.030**	0.060
			Precision	0.251	[0.082, 0.421]	**0.002**	**0.004**
			Recall	0.034	[-0.156, 0.265]	0.406	0.811
	**Qwen 3 4B**	Comprehensive vs Targeted	F1	0.355	[0.256, 0.452]	<0.001	**0.001***
**Center 2 (n=102)**			Precision	0.347	[0.246, 0.449]	<0.001	**0.001***
			Recall	0.367	[0.248, 0.476]	<0.001	**0.001***
		Targeted vs Targeted + SFT	F1	0.123	[0.009, 0.235]	**0.018**	**0.036**

Center	Model	Comparison	Metric	Mean Diff	95% CI	p-value	p-corrected
			Precision	0.192	[0.070, 0.319]	<0.001	**0.001***
			Recall	0.033	[-0.082, 0.148]	0.291	0.582
	**Qwen 3 8B**	Comprehensive vs Targeted	F1	0.202	[0.095, 0.306]	<0.001	**0.001***
			Precision	0.182	[0.068, 0.292]	**0.001**	**0.002**
			Recall	0.228	[0.121, 0.334]	<0.001	**0.001***
		Targeted vs Targeted + SFT	F1	0.048	[-0.044, 0.140]	0.162	0.324
			Precision	0.084	[-0.010, 0.182]	**0.040**	0.080
			Recall	0.003	[-0.096, 0.107]	0.477	0.954
	**Qwen 3 14B**	Comprehensive vs Targeted	F1	0.396	[0.284, 0.511]	<0.001	**0.001***
			Precision	0.388	[0.274, 0.506]	<0.001	**0.001***
			Recall	0.420	[0.298, 0.537]	<0.001	**0.001***
		Targeted vs Targeted + SFT	F1	-0.015	[-0.108, 0.079]	0.628	1.000
			Precision	0.017	[-0.079, 0.120]	0.377	0.753
			Recall	-0.059	[-0.159, 0.055]	0.852	1.000
	**Qwen 3 32B**	Comprehensive vs Targeted	F1	0.254	[0.114, 0.390]	<0.001	**0.001***
			Precision	0.224	[0.081, 0.362]	**0.003**	**0.006**
			Recall	0.316	[0.171, 0.453]	<0.001	**0.001***
		Targeted vs Targeted + SFT	F1	0.054	[-0.058, 0.170]	0.182	0.364
			Precision	0.105	[-0.003, 0.217]	**0.030**	0.059
			Recall	-0.036	[-0.154, 0.087]	0.727	1.000

Statistical Methods: Patient-level bootstrap paired testing with 2,000 iterations. All tests are single-sided (greater than) with Bonferroni correction for multiple comparisons.

Abbreviations: SFT = Supervised Fine-Tuning (LoRA); CI = Confidence Interval; Mean Diff = Mean Difference

Note: Positive values indicate improvement in the second strategy compared to the first.

Supplementary Table 10. Detailed Model Version Specifications

Model Category	Model Name	Version/API	Parameters	Model Type	Access Method	Deployment Platform
**Commercial Models**						
OpenAI	GPT-4.1	gpt-4.1-2025-04-14	Unknown	General	API	Openrouter
OpenAI	O1	o1-2024-12-17	Unknown	Reasoning	API	Openrouter
Anthropic	Claude-3.7-Sonnet	claude-3-7-sonnet-20241022	Unknown	General	API	Anthropic Console
Anthropic	Claude-3.7-Sonnet with think	claude-3-7-sonnet-20250219	Unknown	Reasoning	API	Anthropic Console
Google	Gemini-2.5-Pro	gemini-2.5-pro-exp-03-25	Unknown	Reasoning l	API	Google AI Studio
Google	Gemini-2.5-Flash	gemini-2.5-flash-preview-04-17	Unknown	Reasoning	API	Google AI Studio
Grok	Grok-3	grok-3-beta	Unknown	General	API	xAI Platform
Grok	Grok-3-mini	Grok-3-mini-beta	Unknown	Reasoning	API	xAI Platform
**Open-source Models**						
DeepSeek	DeepSeek-V3	deepseek-chat	671B	General	API	DeepSeek Platform
DeepSeek	DeepSeek-R1	deepseek-reasoner	671B	Reasoning	API	DeepSeek Platform
Alibaba	Qwen3-235B	Qwen3 235B A22B	235B	Reasoning	API	Openrouter
Alibaba	Qwen3-30B	Qwen3 30B A3B	30B	Reasoning	API	Openrouter
Alibaba	QwQ-32B	QwQ-32B	32B	Reasoning	Local	Hugging Face

Alibaba	Qwen3-4B	Qwen3-4B	4B	General	Local	Hugging Face
Alibaba	Qwen3-8B	Qwen3-8B	8B	General	Local	Hugging Face
Alibaba	Qwen3-14B	Qwen3-14B	14B	General	Local	Hugging Face
Alibaba	Qwen3-32B	Qwen3-32B	32B	General	Local	Hugging Face
Google	Gemma-3-27B	Gemma-3-27b-it	27B	General	Local	Hugging Face
Mistral AI	Mistral-Small-3-24B	Mistral-Small-24B-Instruct-2501	24B	General	Local	Hugging Face
**Fine-tuned Models (This Study)**						
Alibaba	Qwen3-4B-PeriComp	Qwen3-4B-PeriComp	4B	General	Local	Hugging Face
Alibaba	Qwen3-8B-PeriComp	Qwen3-8B -PeriComp	8B	General	Local	Hugging Face
Alibaba	Qwen3-14B- PeriComp	Qwen3-14B-PeriComp	14B	General	Local	Hugging Face
Alibaba	Qwen3-32B- PeriComp	Qwen3-32B-PeriComp	32B	General	Local	Hugging Face
Alibaba	QwQ-32B- PeriComp	QwQ-32B-PeriComp	32B	Reasoning	Local	Hugging Face

Note: All models without specific version numbers represent the latest versions available before April 2025

An example of prompt（Translated to English）

Comprehensive original version

You are a senior surgeon. Your task is to identify postoperative complications based on patient data, and the diagnostic criteria for complications are:

1 Acute Kidney Injury

- Definition: Within seven days postoperatively, meeting KDIGO criteria:
  - Grade I: Creatinine 1.5–1.9 times baseline or urine output reduction for 6–12 hours
  - Grade II: Creatinine 2–2.9 times baseline or urine output reduction >12 hours
  - Grade III: Creatinine ≥3 times baseline or requiring renal replacement therapy

2 Acute Respiratory Distress Syndrome (ARDS)

- Bilateral infiltrates within one week of known clinical insult or new/worsening respiratory symptoms, not fully explained by effusions, lobar/lung collapse, or nodules
- Respiratory failure not fully explained by cardiac failure or fluid overload. If no risk factors present, objective assessment (such as echocardiography) is needed to rule out pulmonary edema
- Oxygenation levels:
  - Mild: PaO_2/FiO_2 26.7-40.0 kPa (200-300 mmHg)
  - Moderate: PaO_2/FiO_2 13.3-26.6 kPa (100-200 mmHg)
  - Severe: PaO_2/FiO_2 ≤13.3 kPa (100 mmHg)

3 Anastomotic breakdown

- Definition: Leakage of contents from surgical connection sites, including gastrointestinal, biliary, pancreatic ducts, etc., which may lead to fever, abscess, or organ failure
- Grading:
  - Mild: Asymptomatic, only imaging findings
  - Moderate: Requires treatment, no permanent damage
  - Severe: Requires surgical intervention or causes organ dysfunction

4 Arrhythmia

- ECG-confirmed cardiac rhythm abnormalities
- Grading: Standard grading

5 Cardiac arrest

- Cessation of mechanical cardiac activity with no circulatory signs
- Diagnostic criteria: ECG or clinical examination

- No grading

6 Cardiogenic pulmonary oedema

- Alveolar fluid accumulation due to cardiac dysfunction
- Grading: Standard grading

7 Deep vein thrombosis (DVT)

- Newly formed thrombus in the venous system detected by ultrasound, venography, or CT imaging
- Grading: Standard grading

8 Delirium

- Each criterion met scores one point:
    - Inattention
    - Disorientation
    - Hallucinations-delusions-psychosis
    - Psychomotor agitation or retardation
    - Inappropriate speech or mood
    - Sleep/wake cycle disturbance or symptom fluctuation
- Delirium can be diagnosed when score reaches 4 points
- No grading

9 Gastrointestinal bleed

- Clear clinical or endoscopic evidence of gastrointestinal bleeding
- Grading: Standard grading

10 Infection, source uncertain

- Clinically suspected infection with undetermined source
- Meeting two or more of the following:
    - Core temperature < 36°C or > 38°C
    - White blood cell count > 12 × 10^9 L^-1 or < 4 × 10^9 L^-1
    - Respiratory rate >20 breaths per minute or PaCO2 < 4.7 kPa (35 mmHg)
    - Heart rate >90 beats per minute
- Grading: Standard grading

11 Laboratory confirmed bloodstream infection

- At least one of the following, unrelated to infection at other sites:
    - Positive blood culture with exclusion of other infection sources
    - Clinical symptoms (fever, chills, or hypotension) plus supportive indicators (at least one of the following):
        - a. Common skin contaminants isolated from two or more blood cultures drawn at different times

    - b. Common skin contaminants isolated from intravascular catheter blood culture with appropriate antimicrobial therapy initiated by physician
    - c. Positive blood antigen test
- Grading: Standard grading

12 Myocardial infarction
- Elevated cardiac injury markers (such as TnT) with one of the following:
  - Ischemic symptoms
  - New ECG abnormalities (such as ST changes, left bundle branch block, or pathological Q waves)
  - Regional wall motion abnormalities
- Grading: Standard grading

13 Myocardial injury after non-cardiac surgery (MINS)
- Definition: TnT ≥0.03 ng/mL without other causes (including renal dysfunction)
- No grading

14 Pneumonia
- Must meet all of the following conditions:
  - Radiological criteria (requires two chest X-rays if underlying lung or heart disease, otherwise one is sufficient):
    - New or progressive and persistent infiltrates
    - Consolidation
    - Cavitation
  - Systemic manifestations (at least one):
    - Unexplained fever >38°C
    - White blood cell count <4×10^9/L or >12×10^9/L
    - For patients ≥70 years old, unexplained mental status changes
  - Respiratory-related changes (at least two):
    - New purulent sputum or sputum character changes, or increased secretions/need for more suctioning
    - New or worsening cough, dyspnea, tachypnea
    - Rales or bronchial breath sounds
    - Worsening gas exchange (hypoxemia, need for increased oxygen or ventilator support)
- Grading: Standard grading

15 Paralytic ileus
- Unable to tolerate diet or absence of flatus 3 days postoperatively
- Grading: Standard grading

16 Postoperative haemorrhage

- Blood loss requiring transfusion or surgical hemostasis within 72 hours postoperatively
- Grading: Only includes moderate and severe; surgical hemostasis required is severe

17 Pulmonary embolism (PE)
- Newly formed thrombus in the pulmonary arterial system
- Grading: Standard grading

18 Stroke
- Persistent motor, sensory, or cognitive dysfunction due to embolic or hemorrhagic cerebrovascular events
- Grading: Standard grading

19 Surgical site infection (superficial)
- Infection within 30 days postoperatively
- Limited to incision skin and subcutaneous tissue
- Meeting any of the following:
    - Purulent drainage from superficial incision
    - Pathogenic microorganisms cultured from aseptically obtained fluid or tissue from superficial incision
    - Incision site shows any of pain/tenderness, localized swelling, redness, or heat, and surgeon deliberately opens superficial incision (may be culture positive or not cultured, but culture negative does not meet criteria)
    - Surgeon or attending physician diagnoses surgical site infection
- Grading: Standard grading

20 Surgical site infection (deep)
- Occurs within 30 days postoperatively if no implant, or within 1 year if implant present
- Infection involves deep soft tissues of incision (such as fascia and muscle layers)
- Patient meets at least one of the following:
    - Purulent drainage from deep incision (not from organ/space)
    - Deep incision spontaneously dehisces or is deliberately opened by surgeon, and patient has fever (>38°C) or local pain/tenderness, culture positive or not cultured (culture negative does not meet this criterion)
    - Abscess or other evidence of infection found on direct examination, surgery, pathology, or imaging of deep incision
    - Surgeon or attending physician diagnoses surgical site infection
- Grading: Standard grading

21 Surgical site infection (organ/space)
- Occurs within 30 days postoperatively
- Infection is surgery-related and involves body parts other than skin incision, fascia, muscle layers
- Patient meets at least one of the following:
    - Purulent drainage from drain placed through small incision into organ/space

  - Pathogenic microorganisms cultured from aseptically obtained organ/space fluid or tissue
  - Abscess or other evidence of infection confirmed by direct examination, reoperation, pathology, or imaging of organ/space
  - Surgeon or attending physician diagnoses organ/space surgical site infection
- Grading: Standard grading

22 Urinary tract infection
- Following two conditions appear 24h postoperatively:
  - Positive urine culture (≥10^5 CFU/ml) with no more than two types of microorganisms
  - Simultaneous occurrence of at least one of the following symptoms or signs: fever (>38°C), urgency, frequency, dysuria, suprapubic tenderness, costovertebral angle pain or tenderness, with no other recognized cause
- Grading: Standard grading

JSON Output Format

1. First, output a think field containing an array, where each element is a string representing thoughts and considerations about the case. For example:
   - "Patient recovered well postoperatively with no obvious discomfort"
   - "Elevated troponin T suggests possible myocardial injury"

2. Then, output a complications field containing an array, where each element includes:
   - name: Name of the complication, must be one of the 22 predefined complications
   - grading: Severity grading, can only be one of the following four options:
     - "Mild" - Mild: No treatment required
     - "Moderate" - Moderate: Treatment required, no long-term effects
     - "Severe" - Severe: Causes organ dysfunction or death, significantly prolongs hospital stay
     - "Null" - No grading

Complete format example:
```json
{
  "think": [
    "Patient after kidney transplant surgery, acute kidney injury diagnosis excluded according to guidelines.",
    "First postoperative troponin T elevated to 0.040 ng/mL, meeting definition of myocardial injury after non-cardiac surgery."
  ],
  "complications": [
    {
```

```
            "name": "Myocardial Injury After Non-cardiac Surgery",
            "grading": "Null"
        }
    ]
}
```

Notes:
- If no postoperative complications are found, write "No postoperative complications" in the complications array
- If postoperative complications are found, they must be included in the above 22 complications; custom complications are prohibited
- Standard grading follows:
    - Mild: No treatment required
    - Moderate: Treatment required, no long-term effects
    - Severe: Causes organ dysfunction or death, significantly prolongs hospital stay

Medical Record Data

General Information
- Gender: Male
- Age: 60-65

Progress Notes

2024-05-09 19:06 First Postoperative Progress Note
- Surgery Date: May 09, 2024
- Anesthesia: General anesthesia
- Surgical Procedure: Pancreaticoduodenectomy, retroperitoneal lymph node dissection
- Intraoperative Diagnosis: Pancreatic head cancer
- Postoperative Diagnosis: Pancreatic head cancer
- Surgical Summary: Patient underwent pancreaticoduodenectomy and retroperitoneal lymph node dissection under general anesthesia today. Procedure went smoothly with 600ml blood loss, 400ml fresh frozen plasma transfused. One drain tube placed posterior to biliary-enteric anastomosis and one anterior to pancreatic-enteric anastomosis. Patient returned to ward safely. Gross specimen sent for pathological examination.
- Postoperative Management: Temporary cardiac monitoring, oxygen therapy, NPO with fluid replacement, analgesia. Blood routine and biochemistry to be rechecked tomorrow.
- Important Postoperative Observations: Monitor patient's vital signs and drainage status.
- Recorder: /

2024-05-09 20:17 Automatic Critical Value Progress Note

- 2024-05-09 19:55:31 Emergency biochemistry panel, emergency liver enzyme panel, emergency liver function panel
- Potassium K: 2.71 mmol/L ↓★
- Received Time: 2024-05-09 20:17:39
- Received by: , Processing Time: 2024-05-09 20:17:53, Processed by: , Management: Consistent with condition, must be treated, intravenous potassium supplementation provided.
- Recorder: /

2024-05-10 07:26 Professor/Attending Physician Ward Round

Today is postoperative day 1. Patient complains of wound pain, no fever, chills, chest tightness, shortness of breath, nausea, vomiting or other discomfort. Physical examination: vital signs stable, cardiopulmonary examination shows no obvious abnormalities, abdomen soft, slight reddish drainage from wound, left abdominal drainage 10ml, right abdominal drainage 20ml. Yesterday's urine output 4150ml, intake 8300ml, gastric tube drainage 2ml.

Professor's ward round instructions: Patient complains of significant postoperative wound pain, pay attention to timely symptomatic pain relief; continue liver protection, albumin, gastric protection, nutritional support today, monitor vital signs changes and wound drainage status. Follow orders.

Recorder: /

2024-05-11 08:37 Professor/Attending Physician Ward Round

Today is postoperative day 2. Patient reports wound pain improvement compared to before, oxygen saturation 90-92% without oxygen, can rise to 98% with low-flow oxygen, no fever, chills, chest tightness, shortness of breath, nausea, vomiting or other discomfort. Physical examination: vital signs stable, cardiopulmonary examination shows no obvious abnormalities, abdomen soft, slight reddish drainage from wound, left abdominal drainage 10ml, right abdominal drainage 20ml. Yesterday's urine output 2900ml, intake 3050ml, gastric tube drainage 10ml.

Laboratory Tests:

- Emergency blood routine + high-sensitivity C-reactive protein panel: WBC 12.49x10^9/L ↑, NEUT% 0.868 ↑, Hb 108g/L ↓, PLT 192x10^9/L
- Coagulation studies: PT 13.5 seconds, APTT 29.6 seconds
- Liver and biliary metabolism panel: Na 148mmol/L ↑, CREA 120umol/L ↑, ALT 63U/L ↑, AST 62U/L ↑, ALB 28.1g/L ↓, TBIL 115.6umol/L ↑

Professor's ward round instructions: Patient's postoperative oxygen saturation is decreased, monitor for postoperative cardiopulmonary complications such as pulmonary embolism, may add low molecular weight heparin for thrombosis prevention, recheck relevant tests to exclude complications.

Recorder: /

2024-05-12 07:44 Professor/Attending Physician Ward Round
Today is postoperative day 3. Patient reports wound pain improvement compared to before, oxygen saturation about 90% without oxygen, can rise to 97% with low-flow oxygen, maximum temperature yesterday 37.5°C, no chills, chest tightness, shortness of breath, nausea, vomiting or other discomfort. Physical examination: vital signs stable, cardiopulmonary examination shows no obvious abnormalities, abdomen soft, slight reddish drainage from wound, left abdominal drainage 10ml, right abdominal drainage 10ml. Yesterday's urine output 2065ml, intake 2850ml, gastric tube drainage 10ml.

Professor's ward round instructions: Patient's postoperative gastric tube drainage is low, gastric tube can be removed today, closely monitor condition changes.
Recorder: /

2024-05-15 08:35 Professor/Attending Physician Ward Round
Ward round today, patient has no obvious discomfort, no chills, chest tightness, shortness of breath, nausea, vomiting or other discomfort. Physical examination: vital signs stable, cardiopulmonary examination shows no obvious abnormalities, abdomen soft, no obvious drainage from wound.

Professor's ward round instructions: Patient's postoperative general condition is acceptable, may start feeding and discontinue 3L bag nutritional support, continue monitoring condition changes.
Recorder: /

2024-05-18 07:41 Professor/Attending Physician Ward Round
Ward round today, patient has no obvious discomfort, no chills, chest tightness, shortness of breath, nausea, vomiting or other discomfort. Physical examination: vital signs stable, cardiopulmonary examination shows no obvious abnormalities, abdomen soft, no obvious drainage from wound.

Laboratory Tests: Pathology: (Gross specimen) Tumor infiltrates pancreatic tissue, partial duodenal wall and ampulla, tumor cells show sheet-like infiltration, some in cord-like pattern, abundant cytoplasm, some cytoplasm eosinophilic, some transparent, oval nuclei, focal necrosis, neural bundle invasion and intravascular tumor emboli visible, gastric margin, small bowel margin and bile duct margin show no cancer. Also shows lymph node metastatic cancer (2/2). Specimen 15 immunohistochemistry: Cancer tissue CK7(+), CK19(+), MUC-1(+), CDX-2(-), M-CEA partial(+), INI-1(+, no loss), Brg-1(SMARCA4)(+, no loss), SMARCA2(+, no loss), Syn(-), INSM1(-), Ki-67 about 20%(+). Combined with HE morphology and immunohistochemistry results, lesion consistent with pancreatic poorly differentiated carcinoma, considered adenocarcinoma, suggest additional immunohistochemistry (Trypsin, Chymotrypsin, Bcl-10) to assist in tumor origin analysis.

Professor's ward round instructions: Patient's pancreatic cancer diagnosis is relatively clear, note that postoperative chemotherapy needs to be arranged; patient's postoperative recovery is acceptable, if no obvious discomfort, may consider discharge.
Recorder: /

2024-05-21 08:18 Professor/Attending Physician Ward Round
Ward round today, patient has no complaints of discomfort, good spirit, sleep, appetite, no dizziness, headache, fever, chills, no precordial discomfort, no abdominal distension, abdominal pain, no urinary frequency, urgency, dysuria, no gross hematuria, normal stool and urine color. Physical examination same as before.

Attending physician's ward round instructions: Patient's postoperative recovery is good, may process discharge today. Follow orders.
Recorder: /

Laboratory Results

Test Date	Test Item	Result	Unit	High/Low	Risk Flag	Reference Range
2024-05-04	Creatinine CREA	124	umol/L	↑		53 - 115
2024-05-09	White Blood Cells WBC	11.40	x10^9/L	↑		4.00 - 10.00
2024-05-09	Hemoglobin Hb	103	g/L	↓		130 - 175
2024-05-09	Creatinine CREA	118	umol/L	↑		53 - 115
2024-05-10	Creatinine CREA	120	umol/L	↑		53 - 115
2024-05-10	White Blood Cells WBC	12.49	x10^9/L	↑		4.00 - 10.00
2024-05-10	Hemoglobin Hb	108	g/L	↓		130 - 175
2024-05-12	White Blood Cells WBC	11.78	x10^9/L	↑		4.00 - 10.00
2024-05-12	Hemoglobin Hb	93	g/L	↓		130 - 175
2024-05-12	Creatinine CREA	134	umol/L	↑		53 - 115
2024-05-14	Hemoglobin Hb	101	g/L	↓		130 - 175
2024-05-14	Creatinine CREA	141	umol/L	↑		53 - 115
2024-05-14	High-sensitivity Troponin T (TnT-T) (Luminescence)	0.241	ng/mL	↑		0.000 - 0.014
2024-05-15	Creatinine CREA	159	umol/L	↑		53 - 115
2024-05-16	Hemoglobin Hb	86	g/L	↓		130 - 175
2024-05-16	Creatinine CREA	147	umol/L	↑		53 - 115
2024-05-18	Hemoglobin Hb	90	g/L	↓		130 - 175
2024-05-18	D-dimer (D-D) Test	4.21	mg/L FEU	↑		0.00 - 0.55
2024-05-18	Creatinine CREA	159	umol/L	↑		53 - 115
2024-05-20	White Blood Cells WBC	10.53	x10^9/L	↑		4.00 - 10.00
2024-05-20	Hemoglobin Hb	94	g/L	↓		130 - 175

| 2024-05-20 | Creatinine CREA | 141 | umol/L | ↑ | | 53 - 115 |

# Bacterial Culture

Test Date	Sample	Test Item	Result
2024-05-11	Drainage Fluid	Preliminary Report	Preliminary report: No bacterial growth

# Examination Results

2024-05-13 Imaging Diagnosis

"Radical pancreaticoduodenectomy + retroperitoneal lymph node dissection" follow-up, compared to 2024-05-07 CT:

1. Post-Whipple changes, biliary-enteric, gastro-enteric, pancreatic-enteric anastomoses patent, peritoneal exudate in surgical area, loculated fluid collection, small amount of air, abdominal drainage tubes in place.
2. Lymphatic edema around portal vein. Mild intrahepatic bile duct dilation with small amount of air. Mild pancreatic duct dilation.
3. Multiple abnormal enhancement foci in liver, possibly hemangiomas, suggest correlation with MRI examination.
4. Scattered liver cysts.
5. Multiple bilateral adrenal nodules, possibly nodular hyperplasia, suggest clinical correlation and follow-up.
6. Multiple bilateral renal stones; multiple bilateral renal cysts.
7. Bilateral lower lobe and left lingular segmental atelectasis, bilateral pleural effusion, suggest follow-up.
8. Subpleural inflammation in right middle lobe, bilateral pulmonary emphysema.
9. Coronary artery, aorta and branch atherosclerosis, aortic valve calcification.

2024-05-14 ECG Diagnosis

- Sinus rhythm
- Complete right bundle branch block
- Left ventricular high voltage

Comprehensive modified version

You are a senior surgeon. Your task is to identify postoperative complications based on patient data, and the diagnostic criteria for complications are:

1 Acute Kidney Injury

- Definition: Within seven days postoperatively, meeting KDIGO criteria:

- Grade I: Creatinine 1.5–1.9 times baseline or urine output reduction for 6–12 hours
  - Grade II: Creatinine 2–2.9 times baseline or urine output reduction >12 hours
  - Grade III: Creatinine ≥3 times baseline or requiring renal replacement therapy
- Notes:
  - If the surgery is kidney transplantation, postoperative acute kidney injury should not be diagnosed
  - If there are no preoperative creatinine test results, calculate based on baseline value of 84

2 Acute Respiratory Distress Syndrome (ARDS)

- Bilateral infiltrates within one week of known clinical insult or new/worsening respiratory symptoms, not fully explained by effusions, lobar/lung collapse, or nodules
- Respiratory failure not fully explained by cardiac failure or fluid overload. If no risk factors present, objective assessment (such as echocardiography) is needed to rule out pulmonary edema
- Oxygenation levels:
  - Mild: PaO2/FiO2 26.7-40.0 kPa (200-300 mmHg)
  - Moderate: PaO2/FiO2 13.3-26.6 kPa (100-200 mmHg)
  - Severe: PaO2/FiO2 ≤13.3 kPa (100 mmHg)

3 Anastomotic breakdown

- Definition: Leakage of contents from surgical connection sites (external, drainage site, or imaging findings), including gastrointestinal, biliary, pancreatic ducts, etc., which may lead to fever, abscess, or organ failure
- Grading:
  - Mild: Asymptomatic, only imaging findings
  - Moderate: Requires treatment, no permanent damage
  - Severe: Requires surgical intervention or causes organ dysfunction

4 Arrhythmia

- ECG-confirmed cardiac rhythm abnormalities
- Grading: Standard grading

5 Cardiac arrest

- Cessation of mechanical cardiac activity with no circulatory signs
- Diagnostic criteria: ECG or clinical examination
- No grading

6 Cardiogenic pulmonary oedema

- Alveolar fluid accumulation due to cardiac dysfunction
- Grading: Standard grading

7 Deep vein thrombosis (DVT)

- Newly formed thrombus in the venous system detected by ultrasound, venography, or CT imaging
- Grading: Standard grading

8 Delirium

- Each criterion met scores one point:
    - Inattention
    - Disorientation
    - Hallucinations-delusions-psychosis
    - Psychomotor agitation or retardation
    - Inappropriate speech or mood
    - Sleep/wake cycle disturbance or symptom fluctuation
- Delirium can be diagnosed when score reaches 4 points
- No grading

9 Gastrointestinal bleed

- Clear clinical or endoscopic evidence of gastrointestinal bleeding
- Grading: Standard grading

10 Infection, source uncertain

- Clinically suspected infection with undetermined source
- Meeting two or more of the following:
    - Core temperature < 36°C or > 38°C
    - White blood cell count > 12 × 10^9 L^-1 or < 4 × 10^9 L^-1
    - Respiratory rate >20 breaths per minute or PaCO2 < 4.7 kPa (35 mmHg)
    - Heart rate >90 beats per minute
- Grading: Standard grading

11 Laboratory confirmed bloodstream infection

- At least one of the following, unrelated to infection at other sites:
    - Positive blood culture with exclusion of other infection sources
    - Clinical symptoms (fever, chills, or hypotension) plus supportive indicators (at least one of the following):
        - a. Common skin contaminants isolated from two or more blood cultures drawn at different times
        - b. Common skin contaminants isolated from intravascular catheter blood culture with appropriate antimicrobial therapy initiated by physician
        - c. Positive blood antigen test
- Grading: Standard grading

12 Myocardial infarction

- Elevated cardiac injury markers (such as TnT) with one of the following:

  - Ischemic symptoms
  - New ECG abnormalities (such as ST changes, left bundle branch block, or pathological Q waves)
  - Regional wall motion abnormalities
- Grading: Standard grading

13 Myocardial injury after non-cardiac surgery (MINS)
- Definition: TnT ≥0.03 ng/mL without other causes (including renal dysfunction)
- No grading

14 Pneumonia
- Must meet all of the following conditions:
  - Radiological criteria (requires two chest X-rays if underlying lung or heart disease, otherwise one is sufficient):
    - New or progressive and persistent infiltrates
    - Consolidation
    - Cavitation
  - Systemic manifestations (at least one):
    - Unexplained fever >38°C
    - White blood cell count <4×10^9/L or >12×10^9/L
    - For patients ≥70 years old, unexplained mental status changes
  - Respiratory-related changes (at least two):
    - New purulent sputum or sputum character changes, or increased secretions/need for more suctioning
    - New or worsening cough, dyspnea, tachypnea
    - Rales or bronchial breath sounds
    - Worsening gas exchange (hypoxemia, need for increased oxygen or ventilator support)
- Grading: Standard grading

15 Paralytic ileus
- Unable to tolerate diet or absence of flatus 3 days postoperatively
- Notes:
  - Doctors may forget to record flatus or fasting status. If there are no relevant records at all, gastrointestinal symptoms (such as abdominal distension or vomiting) are required for diagnosis
  - If fasting is due to enterocutaneous fistula, paralytic ileus should not be diagnosed unless there are gastrointestinal symptoms
- Grading: Standard grading

16 Postoperative haemorrhage
- Blood loss requiring transfusion or surgical hemostasis within 72 hours postoperatively

- Notes: If the patient has minimal postoperative drainage with light color but still receives transfusion, consider intraoperative bleeding or pre-existing anemia as the cause; do not diagnose postoperative hemorrhage
- Grading: Only includes moderate and severe; surgical hemostasis required is severe

17 Pulmonary embolism (PE)
- Newly formed thrombus in the pulmonary arterial system
- Grading: Standard grading

18 Stroke
- Persistent motor, sensory, or cognitive dysfunction due to embolic or hemorrhagic cerebrovascular events
- Grading: Standard grading

19 Surgical site infection (superficial)
- Infection within 30 days postoperatively
- Limited to incision skin and subcutaneous tissue
- Meeting any of the following:
    - Purulent drainage from superficial incision
    - Pathogenic microorganisms cultured from aseptically obtained fluid or tissue from superficial incision
    - Incision site shows any of pain/tenderness, localized swelling, redness, or heat, and surgeon deliberately opens superficial incision (may be culture positive or not cultured, but culture negative does not meet criteria)
    - Surgeon or attending physician diagnoses surgical site infection
- Grading: Standard grading

20 Surgical site infection (deep)
- Occurs within 30 days postoperatively if no implant, or within 1 year if implant present
- Infection involves deep soft tissues of incision (such as fascia and muscle layers)
- Patient meets at least one of the following:
    - Purulent drainage from deep incision (not from organ/space)
    - Deep incision spontaneously dehisces or is deliberately opened by surgeon, and patient has fever (>38°C) or local pain/tenderness, culture positive or not cultured (culture negative does not meet this criterion)
    - Abscess or other evidence of infection found on direct examination, surgery, pathology, or imaging of deep incision
    - Surgeon or attending physician diagnoses surgical site infection
- Grading: Standard grading

21 Surgical site infection (organ/space)
- Occurs within 30 days postoperatively
- Infection is surgery-related and involves body parts other than skin incision, fascia, muscle layers
- Patient meets at least one of the following:

  - Purulent drainage from drain placed through small incision into organ/space
  - Pathogenic microorganisms cultured from aseptically obtained organ/space fluid or tissue
  - Abscess or other evidence of infection confirmed by direct examination, reoperation, pathology, or imaging of organ/space
  - Surgeon or attending physician diagnoses organ/space surgical site infection
- Grading: Standard grading

22 Urinary tract infection
- Following two conditions appear 24h postoperatively:
  - Positive urine culture (≥10^5 CFU/ml) with no more than two types of microorganisms
  - Simultaneous occurrence of at least one of the following symptoms or signs: fever (>38°C), urgency, frequency, dysuria, suprapubic tenderness, costovertebral angle pain or tenderness, with no other recognized cause
- Grading: Standard grading

### JSON Output Format

1. First, output a think field containing an array, where each element is a string representing thoughts and considerations about the case. For example:
   - "Patient recovered well postoperatively with no obvious discomfort"
   - "Elevated troponin T suggests possible myocardial injury"

2. Then, output a complications field containing an array, where each element includes:
   - name: Name of the complication, must be one of the 22 predefined complications
   - grading: Severity grading, can only be one of the following four options:
     - "Mild" - Mild: No treatment required
     - "Moderate" - Moderate: Treatment required, no long-term effects
     - "Severe" - Severe: Causes organ dysfunction or death, significantly prolongs hospital stay
     - "Null" - No grading

Complete format example:
```json
{
  "think": [
    "Patient after kidney transplant surgery, acute kidney injury diagnosis excluded according to guidelines.",
    "First postoperative troponin T elevated to 0.040 ng/mL, meeting definition of myocardial injury after non-cardiac surgery."
  ],
  "complications": [
```

```
    {
      "name": "Myocardial Injury After Non-cardiac Surgery",
      "grading": "Null"
    }
  ]
}
```

Notes:

- If no postoperative complications are found, write "No postoperative complications" in the complications array
- If postoperative complications are found, they must be included in the above 22 complications; custom complications are prohibited
- Standard grading follows:
  - Mild: No treatment required
  - Moderate: Treatment required, no long-term effects
  - Severe: Causes organ dysfunction or death, significantly prolongs hospital stay

### Medical Record Data

{Same as the corresponding content in the previous section}

Targeted version (Taking acute kidney injury as an example)

You are a senior surgeon. Your task is to determine whether the patient developed acute kidney injury postoperatively based on the medical record data. The diagnostic criteria for this complication are: **Acute Kidney Injury**

- Definition: Within seven days postoperatively, meeting KDIGO criteria:
  - Grade I: Creatinine 1.5–1.9 times baseline or urine output reduction for 6–12 hours
  - Grade II: Creatinine 2–2.9 times baseline or urine output reduction >12 hours
  - Grade III: Creatinine ≥3 times baseline or requiring renal replacement therapy
- Notes:
  - If the surgery is kidney transplantation, postoperative acute kidney injury should not be diagnosed
  - If there are no preoperative creatinine test results, calculate based on baseline value of 84

Note: All diagnostic criteria must be met (unless the criteria explicitly state that meeting one of them is sufficient). Please output in the following JSON format:

```json
{
  "think": "...(your step-by-step thought process)",
  "bool": "True/False (determine whether the patient developed complications, can only output True or False)",
  "grading": "Null/Mild/Moderate/Severe (severity of the complication, can only output one option)"
}
```

```

### Medical Record Data

**{Same as the corresponding content in the previous section}**
```
